# Low-cost composite autosampler for wastewater sampling

**DOI:** 10.1016/j.ohx.2025.e00631

**Published:** 2025-03-01

**Authors:** Hadi A. Al-agele, Bao D. Nguyen, Liam P. Zimmermann, Gurpreet Singh, Cara Walter, Chet Udell, John S. Selker

**Affiliations:** a OPEnS Lab, Oregon State University, Corvallis, OR, United States; b Department of Biological & Ecological Engineering, Oregon State University, Corvallis OR, United States; c Department of Soil Science and Water Resource, College of Agriculture, Al-Qasim Green University, Al-Qasim District 964, Babylon, Iraq

**Keywords:** Auto-sampler, Composite-sampler, Epidemiology, Low-cost design, Wastewater

## Abstract

Wastewater monitoring is a proven method for detecting viruses, illicit drug consumption, pharmaceutical use/abuse, water pollution, and the occurrence of antimicrobial resistance genes. Analyzing sewage water for pathogens like the SARS-Cov-2 virus could enable early detection of infected areas or virus variants. We developed a low-cost (∼US$1000), open-source, composite automatic water sampler with operational flexibility and simple data logging. Sample quantity is measured and cut off appropriately by continuously weighing the incoming liquid. Our system is customizable in both its hardware and firmware (via options and code modification) which allows for the device to be tailored easily to each application. For example, the user can input sampling quantities from 10 g per sample every 1.5 min to a single 2900 g sample after up to a 16-day delay prior to sampling. The outer diameter and height of the sampler are less than 0.5 m to fit in human-entry openings**.** We present experimental data for sample sizes of 10 g and 100 g at suction heights between 0.65 and 4.75 m.

## Highlights

• The COVID-19 pandemic is detected in wastewater.
• Autosampler was tested with different heights (1.2 m, 3 m, 5 m).
• Testing autosampler with different water volumes such as (10 ml, 100 ml) per cycle.
• Pressure sensor and temperature sensor.
• Load cell.

Specifications table

**Table:** 

Hardware name	OPEnS Micro-Aggregating Sewer Sampler (MASS)
Subject area	Environmental, Planetary and Agricultural Sciences
Hardware type	Field measurements and sensors
Closest commercial analog	ISCO GLS
Open Source License	CERN-OHL-S
Cost of Hardware	$1000.
Source File Repository	https://doi.org/10.5281/zenodo.8333206

## 1 Hardware in context

Wastewater monitoring is important to detect viruses, toxins, and other constituents carried in sewage systems [1], [2], [3], [4], [5]. Wastewater based epidemiology has been employed to detect poliovirus during the global eradication program [6], enteric viruses [7], [8], [9], and the zoonotic hepatitis E virus [10], [11]. A low-cost sampler design is employed to collect water samples from groundwater or sewage, with each design offering distinct properties and characteristics. Jones et al., [12] developed innovative, level-accurate sampling methods for use in existing boreholes, while the Ground Water Protection and Restoration Research Unit (GWPRRU) created and tested a low-cost multiport sock sampler for groundwater monitoring in the UK. The sock sampler design enabled the collection of multiple groundwater samples from specific depths, reaching up to 150 feet (45 m), from individual boreholes in the sandstone aquifer at the field site. Bishop et al., [13] presented a cost-effective dedicated sampling system, where gas-driven water samplers (equipped with vibrating wire piezometers) and saturated zone gas samplers are installed within standard plastic water well casings. They highlighted how multi-level sampling using this relatively low-cost, gas-driven system offers a practical and effective method for studying groundwater contamination, especially when dealing with complex pollutants like chlorinated hydrocarbons. However, our sampler was specifically designed to collect water samples from sewage at depths ranging from 0.65 to 4.75 m below the sampler, with the samples stored at 10 °C to maintain their integrity. Most recently, the COVID-19 pandemic increased the need for affordable wastewater monitoring systems [14], [15], [16], [17], but the price of available systems is over $2500, making them cost-prohibitive in many communities.

Here we provide a new sampler design with capabilities beyond those currently available in terms of sub-sample size repeatability and sampling in low flow conditions, at a lower cost (∼$1000), lighter weight, and similar form factor (48.3 cm in diameter, 53.3 cm in height) to existing samplers (e.g. ISCO GLS and ISCO 3700, Table 1). The OPEnS Micro-Aggregating Sewer Sampler (MASS) design uses a weight-based system to draw sub-samples as small as 10 g + 4 g every 1.5 to 1.75 min allowing compositing of up to 207 subsamples (e.g., one sub-sample every 7 min over a 24-hour period) in the 3000 ml collection bag. In comparison, the ISCO GLS uses a pump revolution counter and liquid detection sensor to collect up to 500 sub-samples as small as 10 ml with a repeatability of 10 ml, a total capacity of up to 9.5 L and a minimum sampling period of one minute or one flow pulse [18].

**Table 1:** Comparison of specifications between the OPEnS Micro-Aggregating Autosampler and the ISCO GLS and 3700.

**Specification**	**OPEnS Micro-aggregating Autosampler**	**ISCO GLS**[18]	**ISCO 3700**[20]
Height (cm)	53.3	67	63.5
Largest diameter (cm)	48.3	42	48.3
Dry Weight (no battery) (kg)	10.5	11.1	16.8
Cooling capacity	9.92 lbs ice and 0.79 Gal bag full of 65°F water: −After 24 h: 51.62 °F (10.9 °C) below ambient −After 48 h: 42.8 °F (6 °C) below ambient	None	20 lbs of ice and 4-gal container full of 65° F water: −After 24 h: 32 °F (18 °C) below ambient −After 48 h 25 °F (14 °C) below ambient (standard thermal resistance factor of R-11)
Power requirement	12 V DC	12 V DC	12 V DC
Intake suction tubing	0 to 7 m, ID 1 cm	1 to 30 m, Vinyl or Teflon® lined, ID 1 or 0.6 cm	1 to 30 m, Vinyl or Teflon® lined, ID 1 or 0.6 cm
Typical line velocity @ Head height	0.32 m/s at 0.95 *m* 0.55 m/s at 0.95 m (24 V) 0.03 m/s at 2.75 *m* 0.03 m/s at 4.4 *m*	0.76 m/s @ 0.9 m; 0.76 m/s @ 3.1 m; 0.58 m/s @ 4.6 *m*	0.76 m/s @ 0.9 m; 0.76 m/s @ 3.1 m; 0.58 m/s @ 4.6 *m*
Smallest sample size	10 g	10 g	10 g
Repeatability	4 g	10 g	10 g
Minimum sample frequency	90 s	60 s	60 s, 1 flow pulse
Maximum sample capacity (L)	3	9.5	9.5 (18.2 with jumbo base)
Maximum lift (m)	6	7.9	7.9
Enclosure	10-gallon cooler	NEMA 4X, 6 (IP67)	NEMA 4X, 6 (IP67)
Sample triggers	Uniform time	Uniform time, flow	Uniform time, non-uniform time, flow, flow paced/time switched, or STORM (time- and flow-paced sampling during sample collection).
Real Time Clock Accuracy	5.25 s per month (at 0–40 °C) [21]	60 s per month, typical	60 s per month, typical
Sample size method	Weight	Liquid presence detector and pump revolution counting	Liquid presence detector and pump revolution counting
Sample retries	Until timer expires (user selectable)	None	Up to 3 attempts (user selectable)

## 2 Hardware description

### 2.1 Overview

The MASS delivers a prescribed weight of water to a 3 L sample collection bag during each scheduled sub-sampling event. Prior to deployment, values such as weight per sample, number of samples, time between samples, and delay prior to the first sample are entered either via a serial monitor interface or in a file on the microSD card. At the time of deployment, the device is activated by switching on power and pushing a button on the top of the electronics enclosure. The sampler has at least a 6 day battery life, fits in most manhole openings, is waterproof from above, costs less than $1000, and meets many of the EPA standards for wastewater sampling [19]: all openings are 6 mm or larger, able to sample at a 20 ft (6.1 m) lift height, purges most of the intake line prior to each sample, uses silicone tubing in the pump and Tygon tubing elsewhere, stores the sample in a Mylar bag, and has a programmable cleaning routine. The sampler does not achieve a greater than 2 ft/s or 0.61 m/s flow velocity, but provides a similar rate of 0.55 m/s at low lift heights (< 1 m) with 24 V provided (Table 1).

### 2.2 Physical and hydraulic setup

**Fig. 1:**
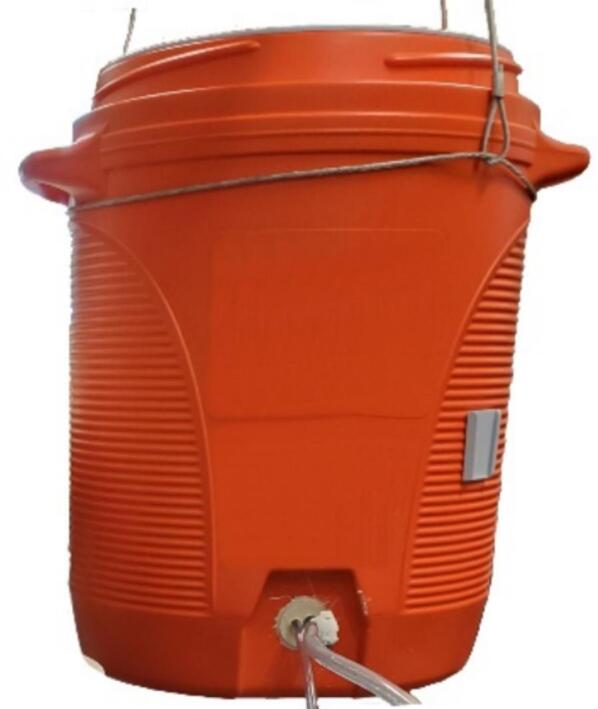
Sampler enclosure showing lid threads, handles, hanging system, and tube entry.

**Fig. 2:**
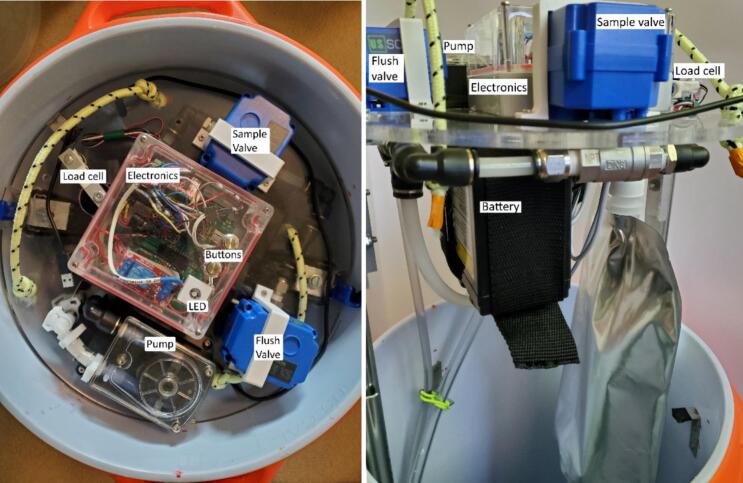
Interior from the top (left) and side (right) of assembled sampler.

The sampler was designed to fit into a 10-gallon water cooler with two heavy-duty handles. The cooler is a one-piece molded shell with a screw-tight lid and spout which we replaced with a sealed tube entry (Fig. 1). With the 3.5 cm overlap between the lid and the body of the cooler, the system is resistant to water entry via splashing and jets directed from the top and side but not from below due to an added drain hole. The system is suspended by three cables with carabiners attached to a cable secured around the cooler under the handles (Fig. 1). Inside the cooler, most of the components are mounted on both sides of a 325 mm diameter laser-cut acrylic disc that rests on right angle brackets attached to the walls near the top of the cooler (Fig. 2). The mechanical lifting system for the disc uses drawer slides to facilitate sample collection and bag attachment and removal. In addition, up to five rigid ice packs (three of 1000 g and two of 750 g) can be mounted inside the cooler.

The main hydraulic related components of the sampler are a peristaltic pump, motorized ball valves, pressure sensor, 3 L mylar bag, and tubing passages at least 6 mm ID to comply with EPA standards for sewage sampling [19] (Fig. 2). The pump has a maximum pumping rate of 3.2 L/min when supplied with 24 V, but was supplied 12 V in this system [22]. The motorized ball valves are normally closed, require 3–5 s to open [23], and operate via a shift register and relay. The piezo-resistive pressure sensor, operating at 3.3 V, monitors the pressure of the inlet tube and turns off the pump and valves when pressure is outside a user-adjustable range relative to starting pressure. The pressure sensor also reports temperature, so the thermal history of the sample is recorded with the data set. For the intake side, to ensure tighter connections, there are barbed fittings and 1 cm inside diameter tubing prior to the pump and for the tubing between the valve and bag connection. All other tubing was 0.6 cm inside diameter with push to connect fittings for ease of replacement.

### 2.3 Electronics

**Fig. 3:**
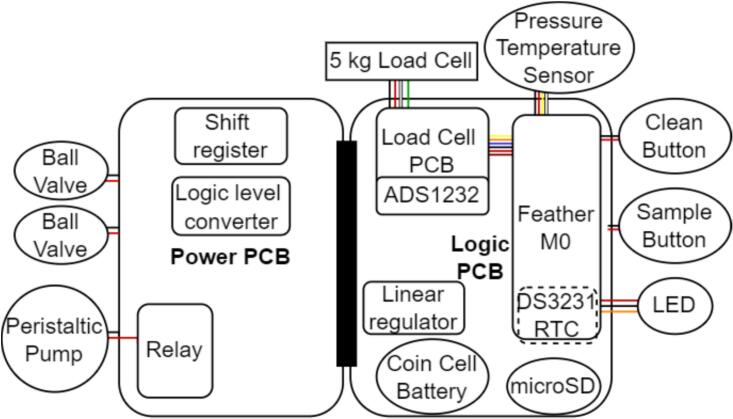
Block diagram of major electrical components for the micro-aggregating sewer sampler. Red lines indicate wired power connections, black lines indicate wired ground connections, and other colored lines indicate wired data connections. (For interpretation of the references to color in this figure legend, the reader is referred to the web version of this article.)

The main electronic components include the power and logic custom printed circuit boards (PCBs) which connect the microcontroller (Adafruit Feather M0 proto), load cell circuit, valves, pump, control relay, buttons, and LED indicator (Fig. 3). The sampler may be powered with any 12 V power source, typically a lead-acid battery.

The Power PCB contains circuitry for connecting and manipulating the valves and pump. The Logic Level converter converts the 3.3 V logic signal to the 5 V signal for the shift register. The shift register controls the ball valves. The circuit was originally designed for an H-Bridge to turn on and off the pump, but an external relay performed better.

The Logic PCB contains circuitry for the real time clock, microSD logging, one button, the buck converter, and a header-based connection for the microcontroller. Protocol timing is set using a DS3231 real-time clock which tracks task time via the coin cell battery even when the battery delivers no 12 V power. The Adafruit Feather M0 controls the relay for the motor, shift-register for valves, and processes sensor data. The clock signals are used via the Feather M0 to change the motor status or open or close the ball valves. The buck converter steps power down from 12 V to 5 V for powering everything except the pump and valves, and has a red LED indicator.

The other button, LED, pressure sensor, and load cell circuit are connected directly to the Feather M0. The two buttons housed on the exterior of the electronics box trigger sampling (ON/OFF) and cleaning (ON/OFF). The LED indicator shows the machine state with simple color coding (Table 2). The pressure sensor is physically connected to the inlet water tube on the off shoot of a T connector to measure the pressure and temperature of water entering the system (Table 3). Data is transferred from the sensor to the microcontroller using the I2C protocol via a shielded 4 conductor 2.5 mm cable that protects the signal from any noise radiating from the peristaltic pump. The load cell circuit measures the sample mass prior to sample initiation, continuously (every 0.08 to 0.12 s) during sampling, and post sample completion. The device requires a 5-kg capacity differential strain gauge four-wire load cell (Table 4). Changes in differential resistance produced by shear force are sensed with an amplified Wheatstone bridge circuit. The load cell is excited by the 5 V power supply from the linear regulator, outputs to an analog-to-digital converter (ADS1232) and then to the Feather M0.

**Table 2:** LED color representation.

Color	State
Blue	No state (waiting for user command)
Green	Running sample
White	Running clean

**Table 3:** Specifications for MS5803 pressure sensor with oversampling ratio of 512 [24].

Sensor	Range	Accuracy	Resolution	Response Time (ms)	Long term stability (mbar/yr)
Pressure (mbar)	10 to 1100	±1.5^a^ or ±2.5^b^	0.084	1.1	±1
Temperature (°C)	−40 to 85	±0.8	<0.01		

a 25 °C, 750 to 1100 mbar.

b -20 °C to 85 °C, 300 to 1100 mbar with autozero at one pressure point.

**Table 4:** Specifications for micro load cell [25].

Weight capacity (kg)	Creep (g/hr)	Zero balance (g)	Cell repeatability error max (g)	Cell non-linearity max, Cell hysteresis max (g)	Temperature effect on span (mg/°C)	Temperature effect on zero (mg/°C)
5 (6 overload)	5	± 75	± 5	5	250	500

### 2.4 Programming

**Fig. 4:**
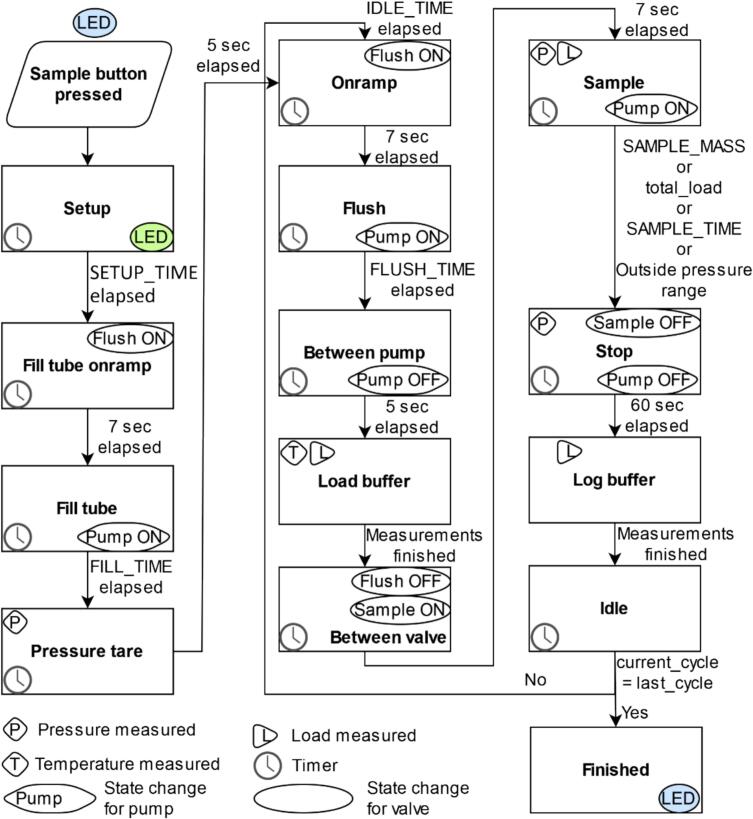
MASS sample state machine diagram. Variables in all capital letters can be changed via the serial monitor or in the state.js file on the microSD card.

Operating parameters of the machine are configured without need to access the source code by connecting the Feather M0 to a computer via USB cable (serial communication) and a serial monitor (e.g. Arduino IDE) or by changing parameters in the state.js file on the microSD card. Variables that can be changed via the serial monitor or state.js file are listed in the Operating Instructions (Section 6). State changes and all measurements are printed to the serial monitor, and time stamped per cycle measurements are saved to the microSD card in the DATA.csv file. The sample state machine diagram is displayed in Fig. 4.

The states in the left column of Fig. 4 occur once upon initiation of sampling either using the sample button or sample button press command in the serial monitor. The setup state delays action for a user-determined period. It is executed at once when the button is pressed. The setup state can be used to schedule the first sampling or to provide the user with time to deploy the sampler after pressing the button. The fill tube onramp and fill tube states open the flush valve and run the pump, drawing water into the tubes at a pressure similar to that of the sample state for the pressure tare. The FILL_TIME variable should be set relative to the height above the sample source and pumping rate (Table 1) to ensure enough time to fill the intake tube. There are 7 s delays at the beginning of each state that opens a valve to allow the valve to fully open. The pressure tare state runs the pump with the flush valve open and prints the mean pressure to the serial monitor and microSD. The upper and lower pressure bounds are computed by adding and subtracting 350 mbar from the mean pressure and include expected fluctuations due to pump action.

The pre-sampling states that occur during every cycle (or sub-sample) are in the middle column of the sample state machine diagram (Fig. 4). The onramp and flush states turn on the flush valve and pump, respectively. The flush state moves old water out of the intake tube, and draws fresh water in, ensuring that the water sampled at any given time is from the source at that time. The tubing between the sample valve and bag is not flushed. The user determines the amount of time the flush state runs (FLUSH_TIME), and it should be set to replace the water in the intake tubing fully. The required flush time can be calculated based upon the average pumping rate by height in Table 1. To reduce noise in the mass measurement, the pump is turned off in the between pump state, and pre-cycle (tare) mass is measured as the average of 25 values along with the temperature during the load buffer state and printed to microSD and serial monitor. Finally, the flush valve is turned off and the sample valve turned on in the between valve state to prepare for sampling.

The sampling, idle, and finished states are in the right column of the sample state machine diagram (Fig. 3). The sampling state starts with the pump turning on, and continues until the first of four conditions are met: the desired per-cycle mass (current mass minus tare mass), total sample mass (2900 g), the maximum sample time elapsing, if the mean of five consecutive pressure measurements is outside of the pressure range, or, after the first 25 measurements, the mass has not increased by at least 0.01 g. Mass and pressure are measured continuously during this state and output to the serial monitor every 0.08 to 0.12 s. When an end of sample state condition is met, the type of condition is printed to microSD and serial monitor. The stop state turns off the pump and valve, and measures and prints to microSD the pressure. There is a delay between the end of sampling and measuring the final mass as an average of 25 values in the log buffer state to avoid noise from the pump and valve actuation. The idle state occurs for the user defined length of time (IDLE_TIME) if all user defined cycles have not been completed and then transitions back to the onramp state for the next cycle or transitions immediately to the finished state which completes the sample.

• MASS provides equal interval and weight composite sampling of up to 3 L of liquids with logging of sample time, individual sample weight, and pressure and temperature at start of sampling. With a network of MASS devices in a sewer environment, locations of water quality or biological components could be attributed to a particular sewer-shed.
• In a surface water environment, MASS could be used for water quality collection across an entire day or multiple days.
• In a food manufacturing setting, with a sample size of as little as 10 g, MASS could be used to verify composition of liquids across a batch.

## 3 Design files

**Table:** 

**Design file name**	**File type**	**Open-source license**	**Location of the file**
Acrylic circle	Dxf	CERN-OHL-S	https://doi.org/10.5281/zenodo.8333206
ADS1232 interface	Brd	CERN-OHL-S	https://doi.org/10.5281/zenodo.8333206
ADS1232 interface	Sch	CERN-OHL-S	https://doi.org/10.5281/zenodo.8333206
Enclosure bottom	Dxf	CERN-OHL-S	https://doi.org/10.5281/zenodo.8333206
Enclosure insert	Dxf	CERN-OHL-S	https://doi.org/10.5281/zenodo.8333206
LED Box	Stl	CERN-OHL-S	https://doi.org/10.5281/zenodo.8333206
LED Lid	Stl	CERN-OHL-S	https://doi.org/10.5281/zenodo.8333206
Logic Module V3	Brd	CERN-OHL-S	https://doi.org/10.5281/zenodo.8333206
Logic Module V3	Sch	CERN-OHL-S	https://doi.org/10.5281/zenodo.8333206
MS5803	Brd	CERN-OHL-S	https://doi.org/10.5281/zenodo.8333206
MS5803	Sch	CERN-OHL-S	https://doi.org/10.5281/zenodo.8333206
Power module mini	Brd	CERN-OHL-S	https://doi.org/10.5281/zenodo.8333206
Power module mini	Sch	CERN-OHL-S	https://doi.org/10.5281/zenodo.8333206
Pressure sensor box	Stl	CERN-OHL-S	https://doi.org/10.5281/zenodo.8333206
Pressure sensor box lid	Stl	CERN-OHL-S	https://doi.org/10.5281/zenodo.8333206
Tube holder	Stl	CERN-OHL-S	https://doi.org/10.5281/zenodo.8333206
Valve bracket	Stl	CERN-OHL-S	https://doi.org/10.5281/zenodo.8333206
Application	Hpp	GNU General Public License v3.0	https://doi.org/10.5281/zenodo.8333206
Clock	Hpp	GNU General Public License v3.0	https://doi.org/10.5281/zenodo.8333206
Constants	Hpp	GNU General Public License v3.0	https://doi.org/10.5281/zenodo.8333206
Button	Cpp	GNU General Public License v3.0	https://doi.org/10.5281/zenodo.8333206
Button	Hpp	GNU General Public License v3.0	https://doi.org/10.5281/zenodo.8333206
LED	Hpp	GNU General Public License v3.0	https://doi.org/10.5281/zenodo.8333206
LoadCell	Hpp	GNU General Public License v3.0	https://doi.org/10.5281/zenodo.8333206
PressureSensor	Hpp	GNU General Public License v3.0	https://doi.org/10.5281/zenodo.8333206
Pump	Hpp	GNU General Public License v3.0	https://doi.org/10.5281/zenodo.8333206
Shell	Cpp	GNU General Public License v3.0	https://doi.org/10.5281/zenodo.8333206
Shell	Hpp	GNU General Public License v3.0	https://doi.org/10.5281/zenodo.8333206
ShiftRegister	Hpp	GNU General Public License v3.0	https://doi.org/10.5281/zenodo.8333206
StateMachine	Hpp	GNU General Public License v3.0	https://doi.org/10.5281/zenodo.8333206
CSVWriter	Hpp	GNU General Public License v3.0	https://doi.org/10.5281/zenodo.8333206
JsonEncodableDecodable	Hpp	GNU General Public License v3.0	https://doi.org/10.5281/zenodo.8333206
CleanStateMachine	Hpp	GNU General Public License v3.0	https://doi.org/10.5281/zenodo.8333206
CleanStates	Cpp	GNU General Public License v3.0	https://doi.org/10.5281/zenodo.8333206
CleanStates	Hpp	GNU General Public License v3.0	https://doi.org/10.5281/zenodo.8333206
SampleStateMachine	Hpp	GNU General Public License v3.0	https://doi.org/10.5281/zenodo.8333206
SampleStates	Cpp	GNU General Public License v3.0	https://doi.org/10.5281/zenodo.8333206
SampleStates	Hpp	GNU General Public License v3.0	https://doi.org/10.5281/zenodo.8333206
main	Cpp	GNU General Public License v3.0	https://doi.org/10.5281/zenodo.8333206
platformio	Ini	GNU General Public License v3.0	https://doi.org/10.5281/zenodo.8333206
state	Js	GNU General Public License v3.0	https://doi.org/10.5281/zenodo.8333206

• **Acrylic circle:** Laser cut platform for attaching components inside the cooler
• **ADS1232 interface (brd):** PCB for interfacing ADC breakout with other circuits
• **ADS1232 interface (sch):** Schematic for PCB for interfacing ADC breakout with other circuits
• **Enclosure bottom:** Drawing for locating and sizing cable gland holes in enclosure bottom
• **Enclosure insert:** Drawing for locating and sizing cable gland holes in enclosure insert
• **LED Box:** 3D printed part to protect the wiring side of the LED
• **LED Lid:** 3D printed part to protect outer side of LED
• **Logic module V3 (brd):** PCB for interfacing RTC, coin cell battery, Feather M0, power PCB
• **Logic module V3 (sch):** Schematic for PCB for interfacing load cell circuit, RTC, coin cell battery, Feather M0, power PCB
• **MS5803 (brd):** PCB for interfacing pressure and temperature sensor
• **MS5803 (sch):** Schematic for PCB for interfacing pressure and temperature sensor
• **Power module mini (brd):** PCB for powering and controlling pump and valves
• **Power module mini (sch):** Schematic for PCB for powering and controlling pump and valves
• **Pressure sensor box:** 3D printed part to protect PCB and wiring for pressure sensor
• **Pressure sensor box lid:** 3D printed part to protect PCB and wiring for pressure sensor
• **Tube holder:** 3D printed part for inlet and outlet tubes to pass through into the cooler. Replacement for spigot.
• **Valve bracket:** 3D printed part to go over the top of each value to hold in place on acrylic circle
• **Application:** Contents of setup and loop for overall code
• **Clock:** Management and functions of real time clock
• **Constants:** Preset values across all devices
• **Button (cpp):** Button function hierarchy
• **Button (hpp):** Button function definitions
• **LED:** LED setup, state and color definitions
• **LoadCell:** Load cell constants, setup, and functions
• **PressureSensor:** Pressure sensor setup and functions
• **Pump:** Pump functions
• **Shell (cpp):** Definition and argument handling for serial monitor entries
• **Shell (hpp):** Class definition for Shell functions
• **ShiftRegister:** Shift register functions
• **StateMachine:** Overall definitions for state machine types of functions
• **CSVWriter:** Function for handling writing strings to SD
• **JsonEncodableDecodable:** Functions for handling json files on SD card
• **CleanStateMachine:** Order of states for Clean State Machine
• **CleanStates (cpp):** Function definitions for Clean States
• **CleanStates (hpp):** Available functions and constants for Clean States
• **SampleStateMachine:** Order of states for Sample State Machine
• **SampleStates (cpp):** Function definitions for Sample States
• **SampleStates (hpp):** Available functions and constants for Sample States
• **main:** File to call setup and loop for overall program
• **platformio:** PlatformIO configuration file
• **state:** Parameters file on the microSD card

## 4 Bill of materials

The bill of materials was provided in the supplementary materials and you can also find them in the link: https://doi.org/10.5281/zenodo.8333206.

## 5 Build instructions

Assembly of this system may also require:

FDM (fused deposition modeling) 3D printer.
Soldering station for soldering sensors and reflow oven for the PCBs.
Laser cutter.
Drill.

### 5.1 3D printed and laser cut parts

Use the design files and an FDM 3-D printer to print, using a material such as ASA (Acrylic Styrene Acrylonitrile), the two valve brackets, the pressure box and box lid, the LED box and box lid, and the tube holder. Follow standard 3D printer safety precautions including not touching the bed or extruder while at high temperatures and keeping away from moving parts. Use a laser cutter, milling machine, or a drill and jigsaw to cut the acrylic sheet into a circle with openings according to the Acrylic_circle design file. Wear appropriate eye and hearing protection for the machine being utilized.

### 5.2 Cooler

The cooler requires five modifications: 1) replace the cooler tap with the 3-D printed tube holder, 2) install 50 mm zinc-plated corner braces, 3) install straps for holding ice, 4) drill and fill a drainage hole in the bottom, 5) attach drawer slides for the lifting system.

**Fig. 5:**
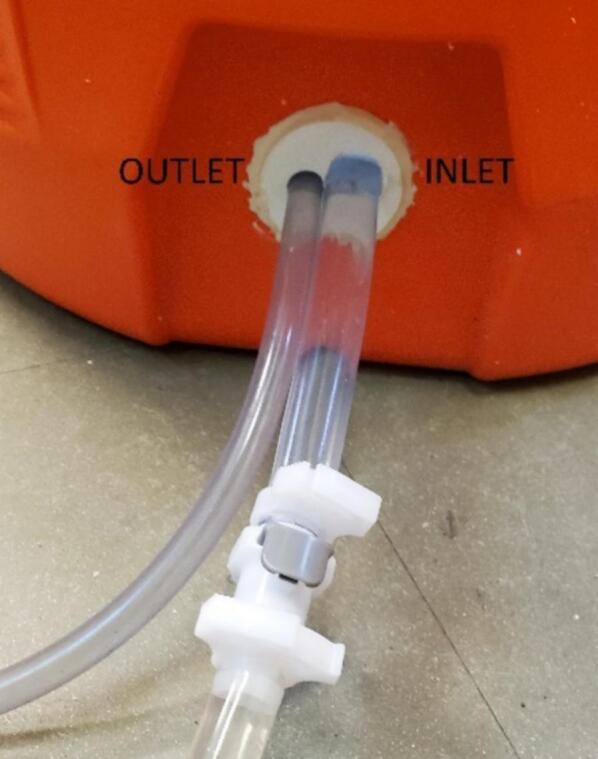
Cooler tap replaced with a 3D-printed inlet and outlet tube holder.

For the first modification, remove the cooler tap, coat the tube holder outside with epoxy, and insert the tube holder into the cooler with the narrower part toward the inside and the tube holes lined up horizontally (Fig. 5).

**Fig. 6:**
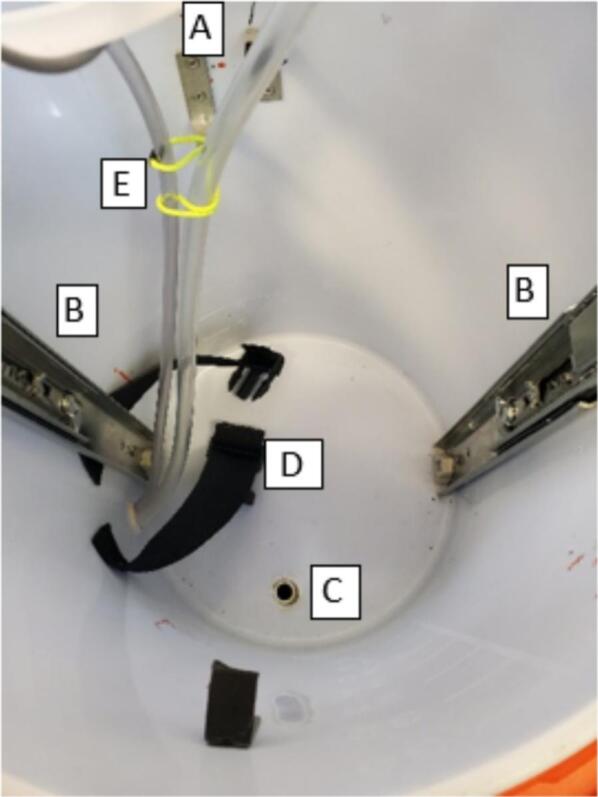
**A)** Interior of the cooler with corner braces, **B**) drawer slides, **C**) drainage holder, **D**) ice pack webbing, and **E**) restrained tubing installed.

Next, cut one leg of each of the two corner braces to a length of 3 cm. Secure the side of the corner brace with two holes with the provided screws to the cooler wall with the top of each bracket 39.5 cm above the bottom of the cooler and approximately across from each other and in line with the left side of each handle (Fig. 6). Optional: install a bracket close to the tube holder upside down as an attachment point for an elastic band to constrain tubing movement inside cooler during acrylic disc lowering (Fig. 6). Wrap tape, e.g. electrical, around the end of each bracket that is sticking out to prevent gouging the sample bag during installation and removal.

For the drainage hole, drill a 2.1 to 2.5 cm diameter hole into the bottom of the cooler 5 cm from the inside wall to allow for drainage in case of lid or hydraulic system failure (Fig. 6). Insert the ½” PVC coupler into the drainage hole, and fill the area between cooler and PVC with glue or epoxy.

**Fig. 7:**
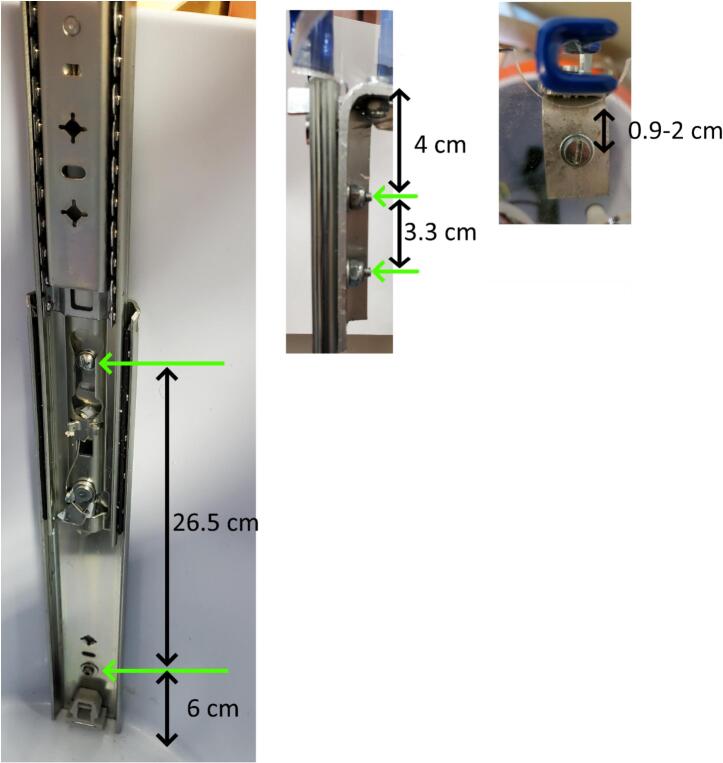
Left: Installed lifting system slide with screw installation heights. Center: Aluminum bracket showing bolt spacing for slide and acrylic circle attachment. Green arrows show bolt locations. Right: Top view of bracket with acrylic circle installed. (For interpretation of the references to color in this figure legend, the reader is referred to the web version of this article.)

For the lifting system, start by extending the drawer slides to their full extent by pulling on the blue handles. Set one of the lifting system slides so that the flat side is resting on the bottom of the cooler 2.5 cm to the right of the side of the tube holder. Check that the bottom of the cooler is level, then use the level to ensure the lifting system slide is fully vertical. Mark the two attachment holes on the cooler, approximately at heights of 6 cm and 31.5 cm above the bottom of the inside of the cooler, remove the slide, and drill 17.48 mm (11/16″) holes at the marks to less than 2.5 cm depth so you do not puncture the outside of the cooler. Fasten the top and bottom of the lifting system slide using the holes existing in the lifting system to the cooler wall with two star drive screws and 5 mm washers (Fig. 7A).

For attaching the slide to the acrylic circle, cut two 2.5 cm wide sections from the aluminum 90° angle to make l-brackets. Cut one leg of each bracket to 3 cm. On the longer leg of each bracket, drill two 5.4 mm diameter holes 3.3 cm apart, centered relative to the narrowest dimension, starting 4 cm below the top of the bracket (Fig. 7B). On the shorter leg, drill one 5.4 mm diameter hole 2 cm from the outside of the longer leg. The single hole to connect the l-bracket to the acrylic circle can be as close at 0.9 cm, but the acrylic circle hole placement would need to be shifted to match. Secure the outside of the sliding end to the long side of the l-bracket with two flathead bolts, washers, and lock nuts (Fig. 7B). Use the blue handle to retract the slide.

To align and attach the other slide, set the acrylic circle on the l-bracket of the installed slide with the holes lined up between the l-bracket and the acrylic circle lifting system attachment hole between the valve cut outs where the nearest valve cut out is to the right of the slide. Fully extend the second lifting system slide, insert into the cooler, and mark the location of the top of the fixed part of the slide on the cooler. Remove the acrylic circle, level the slide while keeping the marked location in place, and mark the two holes. Remove the slide, drill 17.5 mm holes to less than 2.5 cm depth at the marks, insert the slide and fasten to the cooler wall with two star drive screws. Attach the l-bracket to the second slide with two flathead bolts, washers and lock nuts.

To attach three ice packs, secure two pieces of 30 cm long webbing with two of the leftover screws from the corner brace such that the ends are 7 cm apart on either side of the drawer slide next to the inlet/outlet, and the screws are 2.5 cm from the end of each piece of webbing at approximately 15 cm above the bottom of the cooler. Thread the free end of the webbing piece through the two parts of the buckle. Optional additional webbing can be attached with a similar process to secure a medium ice pack perpendicular and to the right of the three ice packs, and to secure a large ice pack perpendicular and to the left of the three ice packs.

### 5.3 Acrylic circle assembly

**Fig. 8:**
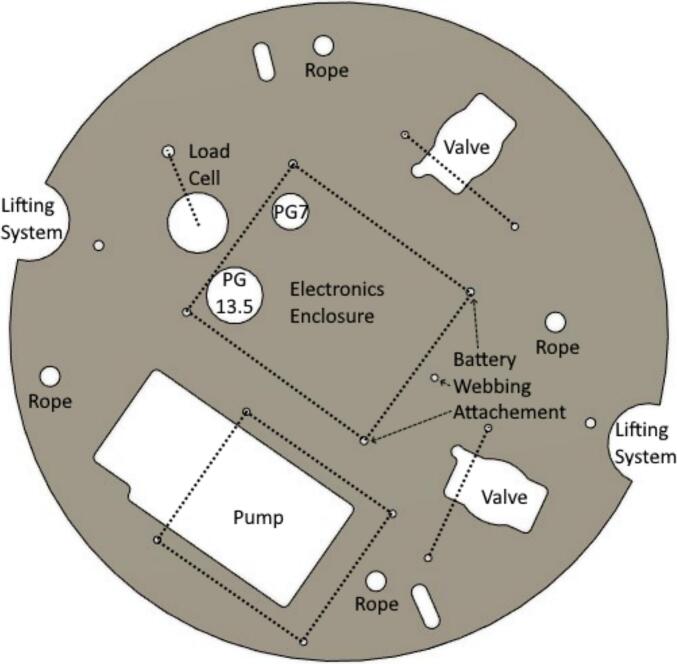
Acrylic circle sized according to the inner diameter of the cooler with labeled component locations. The dotted lines show holes associated with labeled components.

All remaining autosampler parts are arranged on the top or bottom side of the acrylic circle. Cut the acrylic sheet according to Acrylic circle.dxf to fit inside the cooler and provide locations for pass through of materials (Fig. 8). The electronics box, peristaltic pump, load cell, and two motorized valves will sit on top of the acrylic circle (Fig. 8). The battery will be suspended from the bottom of the acrylic circle.

Secure the acrylic circle to the l brackets for the two lifting system slides using flathead bolts, washers and nuts at holes closest to the half circles labeled lifting system in Fig. 8. Raise the lifting system brackets to elevate the acrylic circle out of the cooler for easy access to both sides.

### 5.4 Pump

1. Replace the manufacturer’s tubing: For the pump, it is recommended to replace the provided tubing with the same length (18 cm) 11.1 mm OD pump tubing listed in the bill of materials (Table 5). Remove the top clear cover of the pump by unscrewing the three hex bolts with the provided hex wrench (Fig. 9, left). Slide the tubing end connectors out of the slots, and pull up on the tubing as you manually rotate the pump. Remove the barbed fitting from one side to reuse.

**Table 5:** Pump related tubing and lengths.

**Location**	**Tubing ID (inches)**	**Length (cm)**
Inside pump	5/16	18
Pump intake to barbed tee	3/8	5
Barbed tee to outside cooler	3/8	90
Pump outlet to push to connect tee	¼	42

**Fig. 9:**
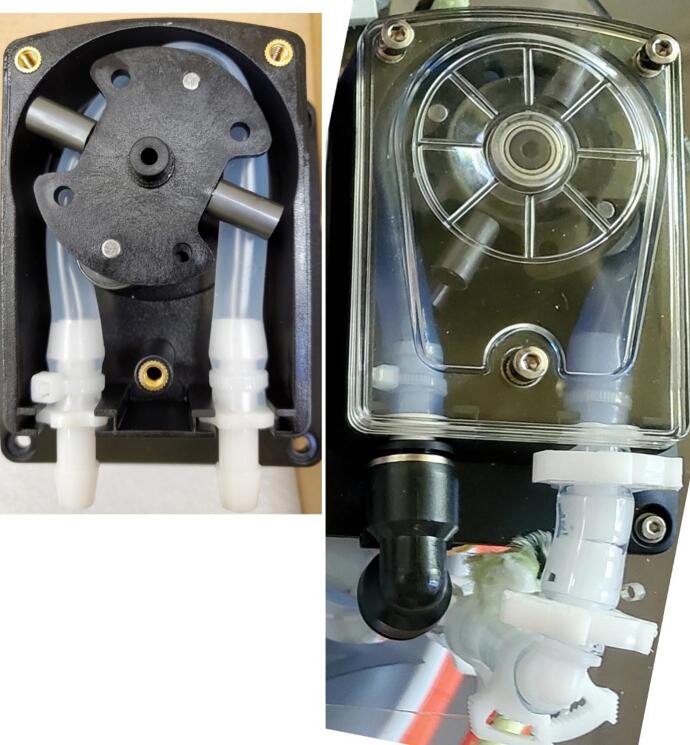
Left: The pump with the original tubing and cover was removed. Right: Pump with replacement tubing and new fittings with outlet on the left and inlet on the right. (https://ar.aliexpress.com/item/4001286485389.html?spm=a2g0s.9042311.0.0.87964c4dVvskfj&gatewayAdapt=glo2ara, 2025). Or the replacement pump from eBay is available online. “12 V/24 V Peristaltic Pump Large Flow Self-Priming Water Liquid Pump Double Roller” (https://www.ebay.com/itm/394990172660?_trkparms=amclksrc%3DITM%26aid%3D1110006%26algo%3DHOMESPLICE.SIM%26ao%3D1%26asc%3D279339%2C279451%26meid%3De13c3d3453394574a9d18d8d2836e769%26pid%3D101875%26rk%3D1%26rkt%3D4%26sd%3D365315786986%26itm%3D394990172660%26pmt%3D1%26noa%3D0%26pg%3D2332490%26algv%3DSimVIDwebV4WithV12Ranker&_trksid=p2332490.c101875.m1851&itmprp=cksum%3A394990172660e13c3d3453394574a9d18d8d2836e769%7Cenc%3AAQAJAAABYGM8MItLMez95oLO7A%252BSOubNMwMt3TwJmfKzLcW52NtuIYujv8KyrSflF1ggdX%252BcwoZ%252F1CbGuq66HNm388HNA4gLiVNai8sVLNjla4yvJn4O7wTxZxGWBetwQxPqrk80dkDSuneoydVtzuuxwmG2Tiwx3Dc%252BcpfRldKmIMtNatxcHKbqk4VZZcSZcUkNyZSoVScU7WUIX0Q38zpKSNIkIuFCDhSCyeLuex7gS6%252BEphAtdu8iVF9uida9doIN8sQcuOiyFRzp5hEdR4nJS%252F93NPNDsv%252FDBiLMcEyjg0d8xjjaHgV%252FrkCWokMWVihwLaaqfO58eLaC8mZArSENZTaaO7y60GBzNRx8B2aqc5oV9gUoc8I1tLQDhmasFe88QrI1LcBejJdewy5h2kKnMI4EPqhvqZeDMh72FpRq5COIGYI4helffrPAAzwCVlIhCIAcad3wsvxHtIEYaGJ6F8e9hew%253D%7Campid%3APL_CLK%7Cclp%3A2332490&itmmeta=01JHSX0S7CS855JR7HF3N3P93Z).
2. Prepare and insert new pump tubing: Cut a length of 18 cm of the new pump tubing. Slide the barbed side of the tube to hose stem fitting into one end of the new tubing and the original barbed fitting into the other end. Cinch a cable tie around the tubing above each fitting and cut off excess. Insert the midpoint disc of the tube to hose stem fitting into the pump tubing exit slot on the outlet (left) side (Fig. 9, right), and manually rotate the pump while pushing in the new tubing until you can put the middle disc of the barbed tube fitting into the other exit slot. Attach a push to connect elbow to the outlet hose stem with the end pointing down toward the pump motor (Fig. 9, right). Cut a 4.4 cm length of the 3/8″ ID tubing, insert a barbed 3/8″ OD elbow into one end, slide on two small snap-grip clamps, and insert the other end of the tubing into the inlet barbed fitting. Slide the snap-grip clamps above the barbs and tighten down (Fig. 9, right). Re-attach the face plate with the three hex bolts (Fig. 9 right).
3. Electrical connections: Solder a 30 cm long black stranded wire to the tab next to the red dot on the motor end (Fig. 10, left). Solder a 30 cm long red stranded wire to the other tab. Note that the pump will rotate counterclockwise with this wiring which corresponds to the tubing setup. Wires can be soldered in the opposite order, but the pump direction and tubing will be reversed. Use electrical tape to fasten the wires up the side of the motor so they exit at the plate (Fig. 10, middle). Wrap the pump motor and wires in two layers of Faraday fabric, using a piece approximately 23 cm by 9 cm, and tape it into place (Fig. 10, right).

**Fig. 10:**
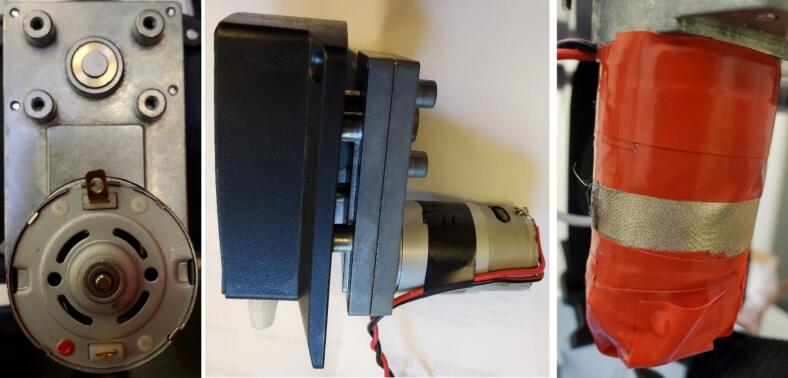
Left: Pump underside showing motor electrical connections. Center: Pump motor with wires taped. Right: Pump motor wrapped in Faraday fabric and taped.
4. Mounting: Set the pump in the acrylic circle at the location shown in Fig. 8, such that the elbows are closest to the load cell location, and slide away from the load cell. The metal part of the pump body, motor, elbows, and wiring will be projected down through the acrylic circle (Fig. 11). Secure the pump to the acrylic circle by inserting four 16 mm long M3 bolts and washers from the top through the pump body and acrylic in each corner of the pump and fastening an M3 nut on the underside of the acrylic.

**Fig. 11:**
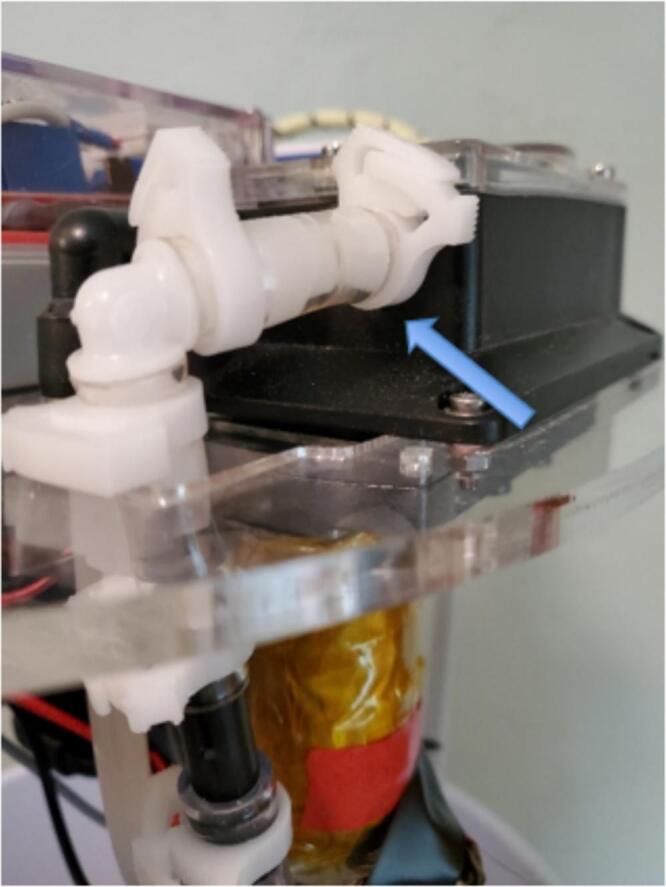
Pump installed on acrylic circle with inlet tubing connections (see arrow).
5. Inlet Tubing: Insert one end of a 5 cm long piece of 3/8″ ID tubing onto the barbed elbow fitting from the pump inlet (Fig. 9, right). Slide two small snap-grip clamps onto the tubing, then insert the other end of the inlet side tubing into one of the two parallel sides of a barbed 3/8″ OD tee fitting. Slide the snap-grip clamps above the barbs and tighten down (Fig. 11). Slide a small snap-grip clamp onto a 100 cm long piece of 3/8″ ID tubing, insert onto the other parallel side of the barbed tee, slide the snap-clamp behind the barbs, and tighten the clamp. Slide through an elastic band (optional) (Fig. 6, center), slide on a small snap-grip clamp, coat the exterior of the tube with epoxy or glue starting 15 cm from the end for 2 cm, and push the end of the tube out the inlet hole of the tube holder until the part covered in epoxy is inside the tube holder (Fig. 5, right). Slide the snap-grip clamp until next to the tube holder on the inside of the cooler, and tighten down to prevent the tube sliding and reduce stress on the seal with the tube holder. Slide a small and then a large snap-grip clamp onto the end of the tube outside the cooler, insert the barbed side of a quick-disconnect tube coupling plug into the end of the tube, slide the large snap-grip clamp over the barbs, and tighten the clamp (Fig. 5, left). Slide the small snap-grip clamp until next to the tube holder, and tighten down to prevent the tube sliding and reduce stress on the seal with the tube holder. Cut the length of 3/8″ tubing needed to reach from the sampler to the liquid to be sampled, slide on a large snap-grip clamp, and insert the quick-disconnect tube coupling socket into the tube. Slide the snap-grip clamp above the barb, and tighten down.
6. Outlet tubing: Insert a 42 cm long piece of polyethylene tubing into the push to connect of the pump outlet and insert the other end into the perpendicular part of a push to connect tee (Table 5).

### 5.5 Valves

1. Port fittings: For the sample valve, wrap two layers of PTFE tape on the threads of a ¼” NPT to 3/8″ straight push to connect fitting, and thread into NPT port on side of valve with the logo on top surface. Wrap two layers of PTFE tape on the threads on an ¼” NPT to 3/8″ barbed elbow fitting and thread into NPT port attachment point (valve oriented with writing right side up) finishing with fitting snug and the elbow horizontal and pointing to the right (Fig. 12 right). For the flush valve, wrap two layers PTFE tape on the threads of the elbow ¼” NPT to 3/8″ push to connect. Thread the NPT to push to connect into the NPT port on the side of valve with the logo on top surface until snug and the open side of the elbow is pointed down. Wrap two layers of PTFE tape on the threads of the ¼” NPT to straight push to connect. Thread the elbow into the other NPT port on the valve until snug (Fig. 12, left). A wrench should be used to finish tightening the fittings.

**Fig. 12:**
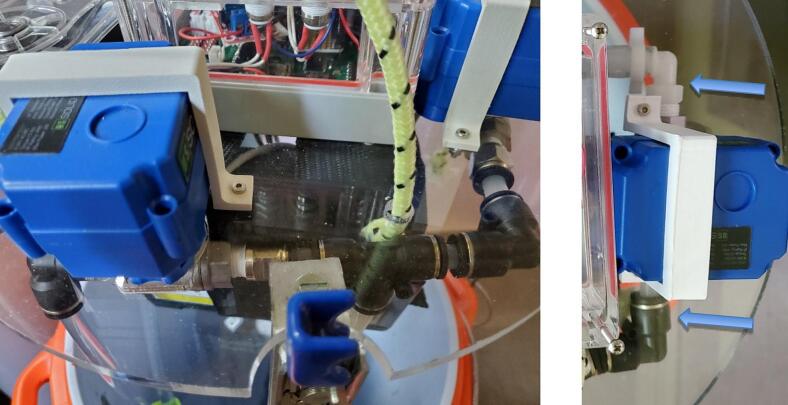
Left: Finished tubing and connections for flush and intake of sample valve. Right: Finished tubing and connections for sample valve.
2. Mounting valves: For each valve, separate the metal hydraulic fittings of the values from the upper part by unscrewing the four small screws from the blue part (Fig. 13). Set the blue top part of the sample valve in the valve location closest to the load cell on top of the acrylic circle such that the U.S. part of the label is closest to the cooler wall (Fig. 8). Make sure the wire cable is sticking out freely underneath (Fig. 13). Insert the hydraulic connection part of the valve into the upper part from below the acrylic circle, and re-attach using the original four screws. Repeat for the flush valve in the location indicated in Fig. 8. Secure each valve with a 3D printed valve bracket, and two 16 mm long M3 bolts, washers and nuts. (Fig. 12).

**Fig. 13:**
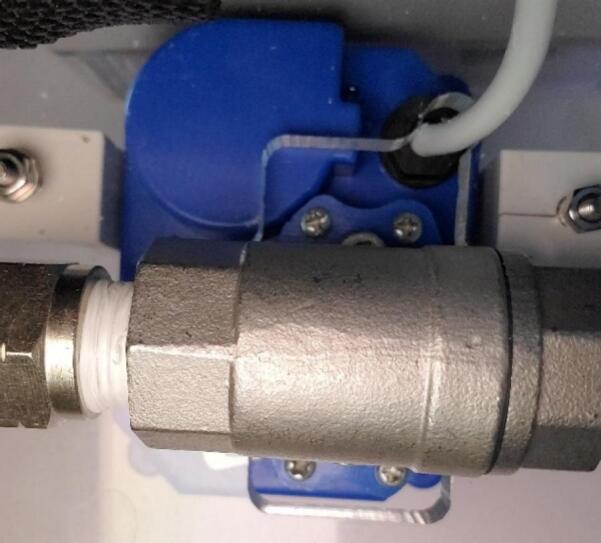
Valve from underneath in finished position showing four screws connecting top and bottom parts of the valve.
3. Sample valve tubing and connections: For the sample valve, slide a large snap-grip clamp onto a 10.5 cm long piece of 3/8″ ID tubing, push one end onto the barbed fitting for the valve, slide the clamp behind the barbs, and tighten the clamp (Table 6, Fig. 12, right). Insert a 6.8 cm long polyethylene tube into the push to connect on the other side of the valve. Insert the other side of the polyethylene tube into an elbow push to connect. Insert a 4.5–5.5 cm long polyethylene tube into the other side of the elbow. Insert the other side of the polyethylene tube from the elbow into one of the parallel sides of a tee push to connect attached to the tubing from the pump outlet.

**Table 6:** Valve related tubing lengths.

Location	Tubing ID and Material	Length (cm)
Sample valve to bag connector	3/8″ PVC	10.5
Sample valve to elbow	¼” polyethylene	6.8
Sample valve side elbow to tee	¼” polyethylene	4.5–5.5
Tee to flush valve	¼” polyethylene	4.5–5.5
Flush valve to cooler outlet	¼” PVC	80
4. Flush valve tubing and connections: Insert one side of a 5.7 cm long polyethylene tube into the straight push to connect of the flush valve and then the other side into the open port of the push to connect tee connected to the pump and sample valve. Insert at least a 80 cm long ¼” ID PVC tube into the elbow push to connect on the other side of the valve, and thread the elastic band from the inlet tube through the corner bracket then thread the tube through it (optional). Coat the outside of the tubing 10 cm from the end, the length that will be outside the cooler, with epoxy and push through the outlet hole of the tube holder until the epoxy section is inside the tube holder. If not using an elastic band and a corner bracket to constrain the inlet and outlet tubes, they can alternatively be cable tied together for a similar result. Insert the end of the tube outside the cooler into a straight push to connect fitting.

### 5.6 Load cell attachment to acrylic circle

**Fig. 14:**
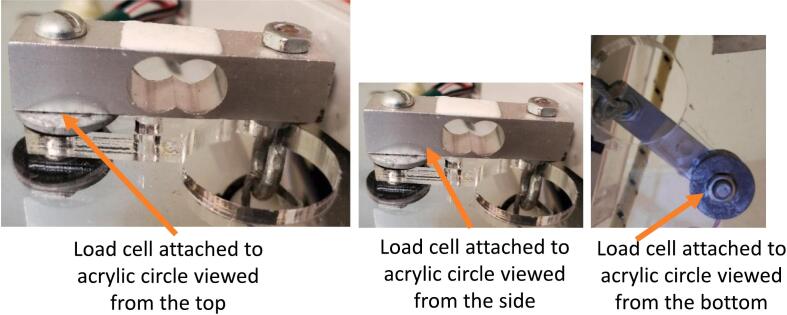
Left: Load cell attached to acrylic circle viewed from the top. Center: Load cell attached to acrylic circle viewed from the side. Right: Load cell attached to acrylic circle viewed from the bottom.

Place the load cell on top of the acrylic circle in the location indicated in Fig. 8 with the mounting hole adjacent to the wire exit above the small hole in the acrylic, and the other hole above the large hole in the acrylic. Make sure the arrow on the side of the load cell is pointing down. Under the load cell above the small hole in the acrylic, stack the spacer, neoprene washer, and a large washer (Fig. 14, middle). Insert a 40 long mm M5 bolt through top of the load cell, washers, spacer, and the acrylic circle (Fig. 14). Under the acrylic circle, slide on another neoprene washer, large washer, and M5 nut. Tighten the M5 nut until snug.

### 5.7 Bag attachment

**Fig. 15:**
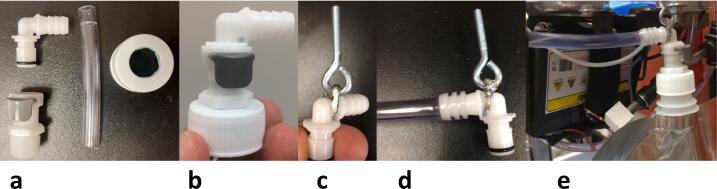
A) Bag cap with hole; B) Coupler threaded into bag cap viewed from the side; C) Coupler threaded into bag cap viewed from below. D) Elbow coupler connected with tube; E) Elbow coupler connected to hook with aluminum cable.

Drill a 1.5 cm diameter hole in the bag cap (Fig. 15A) and thread into the bag cap (Fig. 15B, 15C). Attach the open end of the 10.5 cm long piece of 3/8″ ID tubing from the valve to the barbed side of the elbow coupler (Fig. 15D, Table 6). Thread a 6.5 cm long piece of 1/16″ wire rope through the hole in the elbow coupler just past the collar on the barbed side and through the eye bolt. Slide a crimp onto both ends of the cable, and crimp down (Fig. 15E). Fasten the eye hook to the open load cell hole from the bottom with the provided nuts on either side of the load cell (Fig. 14 left).

### 5.8 Electronics box

**Fig. 16:**
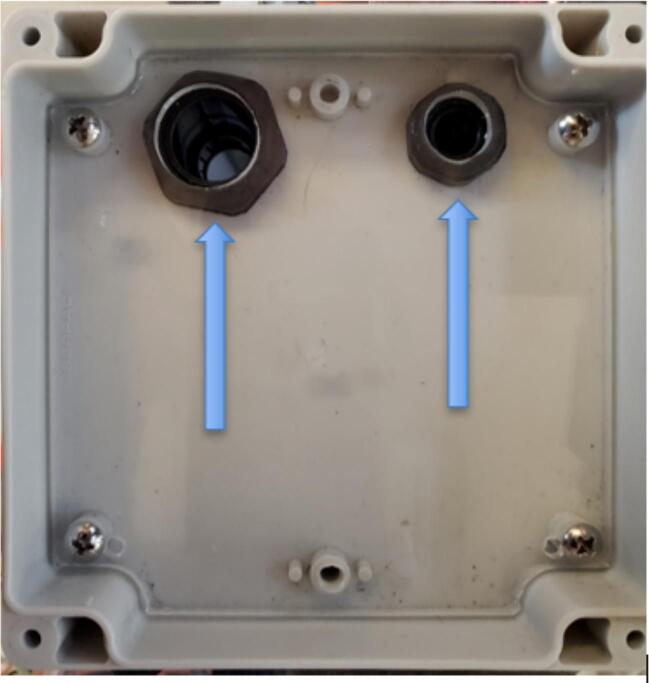
Enclosure bottom and insert showing drilled holes and cable glands.

For the electronics box bottom, following the enclosure bottom drawing, drill one 12.3 mm diameter hole centered 18.6 mm from the closest edge and 34.5 mm from the other edge along one of the sides with an extra internal mounting hole (Fig. 16). Along the same side, drill one 20 mm diameter hole centered 50 mm from the first hole, and 20.5 mm from the box outer edge. Following the enclosure insert drawing, drill matching holes in the enclosure insert: one 12.3 mm diameter hole centered 14.3 mm from the closest edge and 32.8 mm from the other edge, and one 20 mm diameter hole centered 50 mm from the first hole and 16 mm from the edge (Fig. 16).

In the box bottom, insert from the bottom a PG7 cable gland in the smaller hole and a PG13.5 cable gland in the larger hole such that the glands project down outside the box and fasten each on the inside of the box with the supplied nut. Set the enclosure insert inside the box and fasten with provided screws. Set the box bottom on top of the acrylic circle in the location indicated in Fig. 8 with the side with cable glands closest to the load cell location to line up with the larger holes in the acrylic circle. Fasten the box to the acrylic with 2 star drive screws using the pre-drilled holes only on the side with the cable glands. The other two screws will be fastened with the webbing for attaching the battery.

**Fig. 17:**
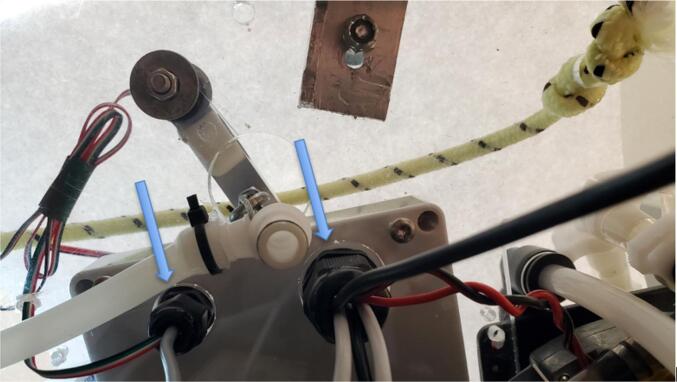
Cables entering cable glands for electronics enclosure from bottom.

Feed the bare wire end of the power plug through the large cable gland from the top so that the plug is inside the box and the bare wires are below (Fig. 17). Feed the micro end of the micro USB cable through the large cable gland from the bottom so that the A side is outside the box and the micro side is inside. Feed the A side of the cable up through the one of the oval holes in the acrylic circle so the end is accessible. Feed the black and red wires attached to the pump up through the large cable gland into the electronics box. Feed the cables from the two valves into the box from the bottom through the large cable gland. Cut two sets of 2 pin JST socket wires down to 3 cm. Slide a solder seal connector onto each of the valve wires. Twist together the red to red and black to black wires for each valve to each set of JST wires. Slide each solder seal connector so that the silver ring of solder is over the wire connection, and use a heat gun to melt the solder and seals. Insert the four wire ribbon cable from the load cell down through the adjacent hole in the acrylic circle and up through the PG7 cable gland into the electronics box.

**Fig. 18:**
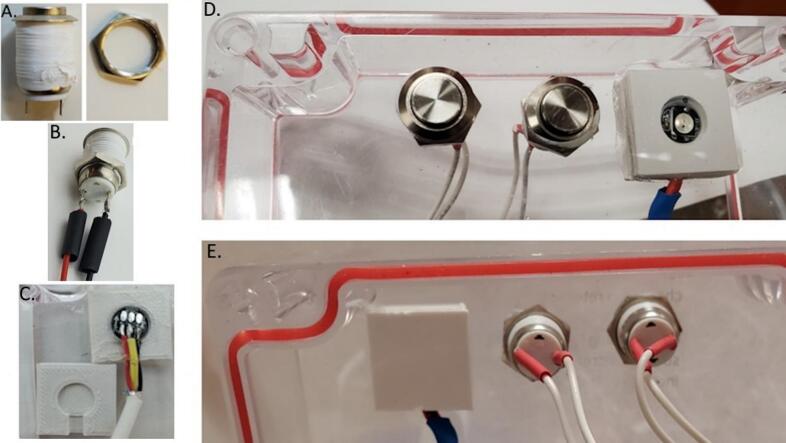
A. Button with wrapped threads and separate nuts. B. LED, box, and lid. C. Electronic box lid from the top with the Clean button, Sample/Stop button, and LED status indicator. D. Electronic box lid from the back.

The sample and clean buttons need wires with JST connectors soldered onto each of the tabs and are then inserted through the lid of the enclosure box. For each button, take the nut off the sample and clean buttons, and wrap PFTE tape around the threads (Fig. 18A). Slide 1.5 cm long pieces of 3 mm diameter heat shrink over each bare wire end of the two female JST wires (Fig. 18B). Orient the button so that the hole with the + symbol is on the left, and solder the red (positive) wire to the top button tab. Solder the black (ground) wire to the bottom button tab. Cover the exposed terminals and soldered wire with the small heat shrink and use a heat gun to shrink it (Fig. 18E). Drill two 12 mm diameter holes in the lid spaced 2.5 cm apart and 2 cm from the box lid perimeter. Insert the buttons in the two holes from the outside of the box and fasten with included nuts on the backside of the box lid (Fig. 18D, E).

For the LED, cut off a 15 cm long piece of the 3-wire cable, and strip back a length of 2.5 cm of the outer jacket, and a length of 3–4 mm of each wire jacket. Slide a 1.5 cm long 4 mm diameter heat shrink over the cable. Solder the exposed ends of the three wires onto the LED pads on the side with the Din label with the black wire to gnd (−), yellow to the middle pad, and red wire to 5 V (Fig. 18C). Slide the 3 mm diameter heat shrink over the soldered connection, and heat to shrink (Fig. 18D). For the other end of the cable, use solder seal connectors to connect the black, yellow and red wires to male jumper wires (cut in half and jacket stripped back 1 cm on one bare end). Insert the LED from the inside facing out into the LED lid, add super glue on the lid inside and set on the bottom. After bottom and top are held together, super glue the LED lid into place on the inside of the box lid (Fig. 18D).

### 5.9 Battery attachment

**Fig. 19:**
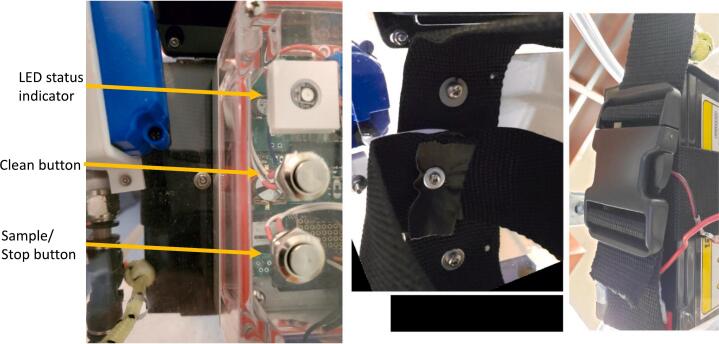
Left: Battery fastener shown from the above the acrylic circle. Center: Battery fasteners shown from below the acrylic circle. Right: Battery shown fastened in place.

The battery holder consists of three pieces of webbing: one 35 cm long that will be a closed loop, and two 25 cm long that will fasten together with a buckle. Make a 5 mm diameter hole in both ends of the 35 cm long webbing at least 1.5 cm from the cut end and centered in terms of narrow dimension of the webbing. Attach both ends of the 35 cm long piece to the acrylic circle using a 16 mm long M3 screw, two M3 9 mm washers (one above and one below), and M3 nut in the hole just outside the enclosure boundary between the enclosure and flush valve (Fig. 19). Make a 5 mm diameter hole in one end of each of the 25 cm long pieces of webbing at least 1.5 cm from the end and centered in terms of narrow dimension of the webbing. Attach each 25 cm piece with a star drive screw and 5 mm washer through the acrylic circle and enclosure box (Fig. 19 center). Thread the buckle pieces through the opposite webbing ends.

For the battery wiring, slide on, crimp and heat shrink a quick-disconnect terminal onto each bare wire from the power plug. Attach the terminal for the red wire onto the red terminal of the battery, and the terminal for the black wire onto the black terminal of the battery. Slide the battery into the webbing loop with the battery terminals on the non-pump side. Wrap the webbing pieces with buckles around the battery perpendicular to the loop, attach the buckles, and tighten (Fig. 19, right).

### 5.10 Electronic circuits

#### 5.10.1 Power module

**Fig. 20:**
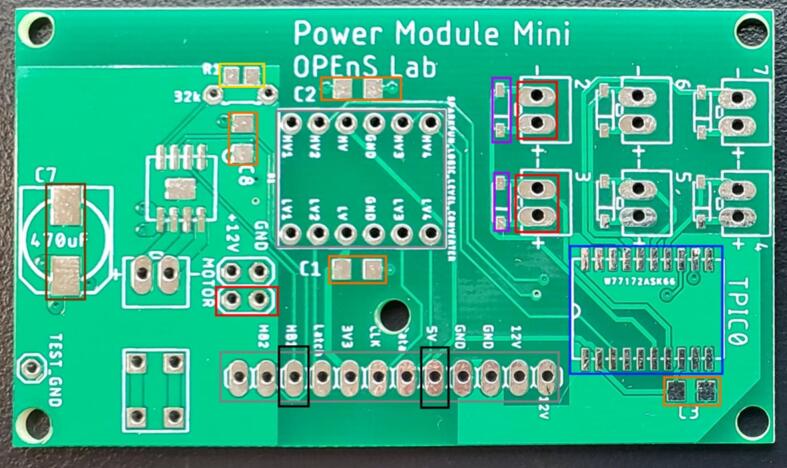
Power Module PCB front without components. See Table 7 for a description of box colors.

**Fig. 21:**
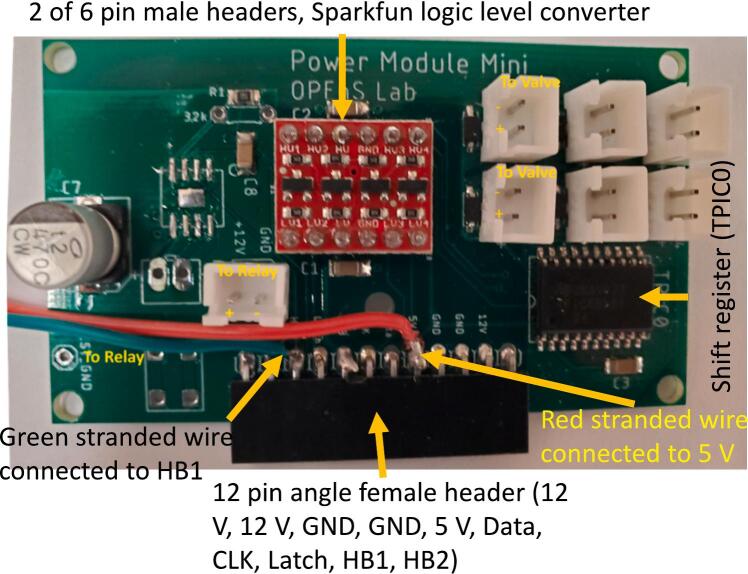
Power module PCB front fully populated. Note that only two of the 2 pin JST connectors and diodes at the right of the board need to be populated for this device, and there are alternatives noted in the text for the green and red wires. (For interpretation of the references to color in this figure legend, the reader is referred to the web version of this article.)

Following Fig. 20, Fig. 21, Table 7, and the Power Module Mini design file, use solder paste and a reflow gun or oven to add the SMD components then hand solder the through hole components to the front of the power module PCB. Cut the set of 12 pin male headers from the Feather M0 into two lengths with 6 pins. Solder the two sets of 6 pin male headers to the power module PCB on one side and then the logic level converter on the other side. Solder the male leads of the 12-pin angle female header to the PCB in the holes starting with the one labeled 12 V such that the open part of the header is facing away from the board (Fig. 21). Solder an 8 cm long red stranded wire to the exposed lead of the 12-pin header labeled 5 V (Fig. 21). Solder an 8 cm long green stranded wire to the exposed lead of the 12-pin header labeled HB1. Alternatively, the HB1 wire can instead be a male jumper wire directly on the Feather M0 to the 6-pin, and the 5 V can be soldered to the far right labeled through hole on the logic PCB. No components are added to the back of the PCB.

**Table 7:** Description, board labels, color coding, type of component, and orientation for power module PCB components.

**Part description**	**Board label**	**Figure color code**	**Type**	**Orientation**
100nF capacitor	C1, C2, C3, C8	Orange box	SMD	Any
470 uF capacitor	C7	Brown box	SMD	Match dog ears on PCB silk screen
32 K resistor	R1	Yellow box	SMD	Any
Flyback Diode for valves	D (D25, D26)	Purple box	SMD	Match offset line between pads from silk screen
JST XH 2 pin	motor, 2, 3	Red box	Through hole	Motor: Cut out toward header; 2, 3: cut out toward logic level shifter
Shift register	TPIC0	Blue box	SMD	Dimple bottom left corner
2 of 6 pin male headers	Sparkfun logic level converter	Light gray box	Through hole	Any
Logic level shifter	Sparkfun logic level converter	Light gray box	Through hole	Text right side up matching board text
12 pin angle female header	12 V, 12 V, GND, GND, 5 V, Data, CLK, Latch, HB1, HB2	Gray box	Through hole	Open side pointing away from board
Red stranded wire, green stranded wire	5 V, HB1	Black box	Through hole	Any

#### 5.10.2 Relay

**Fig. 22:**
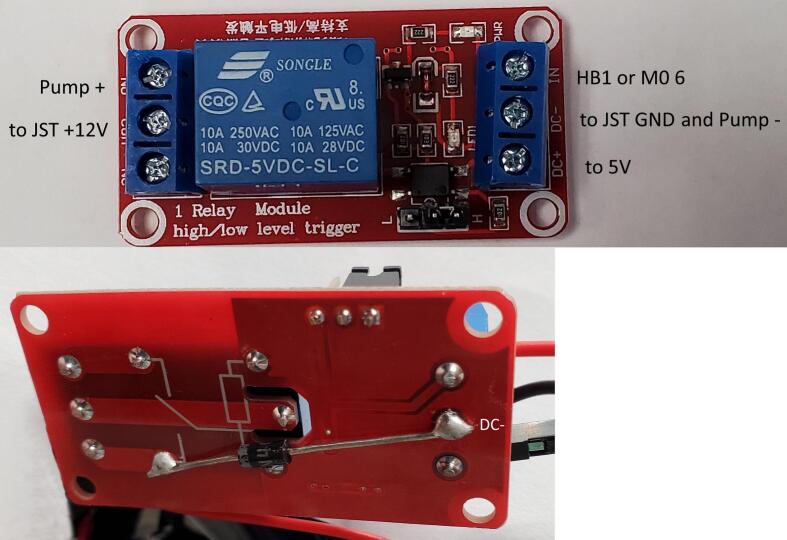
Relay PCB. Top: Front with labeled ports. Bottom: Back with diode connecting ground (DC-) to relay module.

On the back of the relay pcb, solder a rectifier diode from the DC- to the left bottom pin of the relay (Fig. 22 bottom). Insert the bare end of the black wire from a female two pin JST and the black (negative) wire from the pump into the DC- terminal and tighten down (Fig. 22 top). Insert the bare end of the red wire from the female two pin JST into the COM terminal and tighten down. Insert the JST connector into the power PCB ensuring that the red wire is going to the + 12 V side and the black wire is going to the GND side (Fig. 21). Insert and tighten down into the IN terminal either the wire soldered to the HB1 header on the power PCB (Fig. 21) or a male jumper and later insert the other end into the 6 pin on the Feather M0 stacking header. Insert and tighten down the red (positive) wire from the pump into the NO (normally open) terminal (Fig. 22). Insert and tighten down the other end of the wire soldered to the 5 V header on the power PCB (Fig. 21).

#### 5.10.3 Logic module

**Fig. 23:**
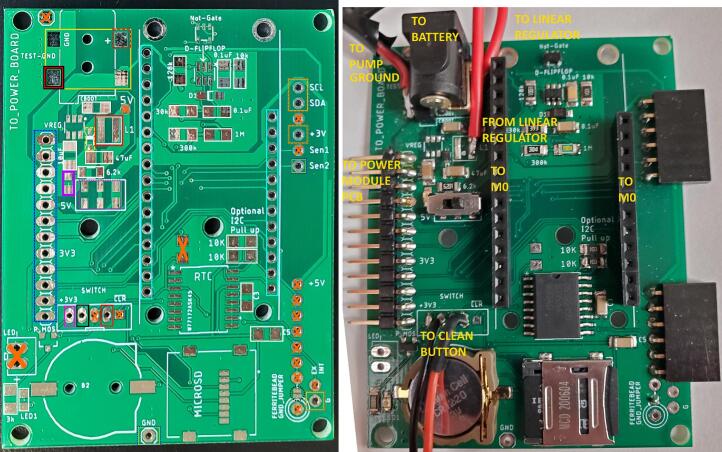
Top of logic module PCB. Left) Unpopulated. See Table 8 for a description of box colors. Pads or holes with orange Xs are not populated. Right) Populated with labels. Note that connections for an external linear regulator are shown instead of the SMD linear regulator and inductor due to part availability, and unused female angle headers are attached on the right side of the board. (For interpretation of the references to color in this figure legend, the reader is referred to the web version of this article.)

Following Fig. 22, Fig. 23, Table 8, and the Logic Module design file, use solder paste and a reflow gun or oven to add the SMD components then hand solder the through hole components to the front of the logic module PCB. Use solder to connect the two pads in the lower right corner of the front of the PCB (circled in dotted black in Fig. 22 left). Optionally, the free ends of the 3 conductor cable wires from the LED can be soldered to a ground through hole (black wire), the Sen2 through hole (yellow wire), and the + 3 V through hole on the right side either directly or with an intermediate connector (Table 8). If also soldering on the wiring for the pressure sensor, wait to solder the + 3 V wire with the pressure sensor power wire as well. On the back of the PCB, solder the 10 K through hole resistor into the holes outlined in red and pink (+3V3) under the “Switch” label in Fig. 22 left. The voltage regulator this PCB was designed to use became unavailable during construction so a linear regulator is shown hardwired in Fig. 22. Insert the coin cell battery into the coin cell battery holder and the microSD card into the microSD mount on the logic module PCB (Fig. 22 right).

**Table 8:** Description, board labels, color coding, type of component, and orientation for logic module PCB components.

**Part description**	**Board label (in design file)**	**Figure color code**	**Type**	**Orientation**
Feather female headers	No label	Light blue	Through hole	Any
Angle male header	No label	Dark blue	Through hole	Short end in board, long end outside board
MicroSD mount	MICROSD	White	SMD	Writing rotated 90° counter-clockwise
Coin cell battery holder	B2	White	SMD	Match silk screen shape
Barrel jack	No label (12 V IN)	Solid Yellow	SMD	Jack opening at board edge
Switch	No label (U$1)	Dotted Pink	SMD	Writing right side up and on right side
RTC	RTC	White	SMD	Dimple at top
P-channel MOSFET	P-MOS	White	SMD	Match pads to pins
Not-Gate	Not-Gate (U$3)	White	SMD	Match pads to pins
D Flipflop	D-FLIPFLOP	White	SMD	Pin 1 next to silk screen *
Zener Diode	D1	None	SMD	Match offset line in silk screen
Linear regulator	VREG	None	SMD	Pin 1 next to silk screen dot
Inductor	L1	None	SMD	Dot farthest from L1 marking
Diode Schottky	No label (D3)	Dashed yellow	SMD	Match offset line in silk screen
Red LED	No label (LED2)	Purple	SMD	Match offset mark in silk screen to indicator on LED
Yellow LED	LED1	None	SMD	Match offset mark in silk screen to indicator on LED
3 K resistor	3 K (R4)	White	SMD	Any
6.2 K resistor	6.2 K (R11)	White	SMD	Any
10 K resistor	10 K (R3, R5, R8)	White	SMD	Any
39 K resistor	30 K (R7)	White	SMD	Any
100 K resistor	128 K (R6)	White	SMD	Any
300 K resistor	300 K (R2)	White	SMD	Any
1 M resistor	1 M (R1)	White	SMD	Any
0.1uF/100 nF 1206 capacitor	0.1uF (C1, C2, C3, C5, CBOOT)	White	SMD	Any
10uF 1206 capacitor	10uF (CIN)	White	SMD	Any
47uF 1206 capacitor	47uF (COUT)	White	SMD	Any
10 K through hole resistor	Switch: 3 V, no label	Pink, Red	Through hole	Any
2 pin JST wires − male	Switch: no label	Black, Red	Through hole	Any
Pressure sensor wires (solder option)	SCL, SDA, +3 V, G	Yellow, dashed orange	Through hole	Any
LED wires (solder option)	GND, Sen2, +3 V	Yellow, dashed blue; Yellow, dashed orange	Through hole	Any

**Fig. 24:**
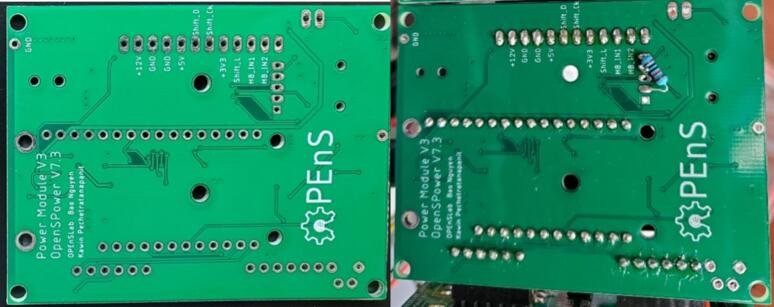
Bottom of logic module PCB. A) Unpopulated. B) Populated.

In Fig. 22 and Fig. 23, the push button is connected between GND (pin 4) and WAKE_NOSLEEP (pin 2). The pin's naming does not reflect its actual purpose but is due to the reuse of a PCB from a previous project. The 10 k through-hole resistor connects WAKE_NOSLEEP and 3.3 V (pin 5) to provide a pull-up when the button is not pressed (Fig. 24).

#### 5.10.4 Load cell circuit

**Fig. 25:**
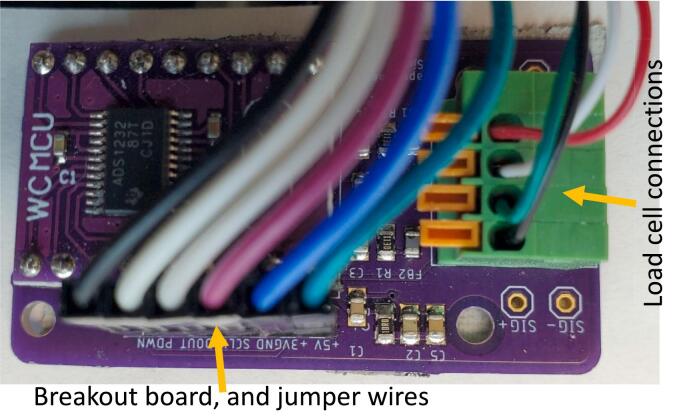
Populated load cell PCB. Left: Load cell connections only. Right: Load cell connections, breakout board, and jumper wires.

Following Fig. 25, Table 9, and the ADS1232 interface design file, use solder paste and a reflow gun or oven to add the SMD components then hand solder the through hole components to the front of the ADS1232 interface PCB. Solder the two sets of 9 pin male headers that came with the ADS1232 PCB onto the board such as the spacer and long side are against the side of the board with the hole labels. Insert the headers from the ADS1232 PCB into the two rows of 9 pin female headers on the ADS1232 interface board such that the 3.3 V and 5 V pins on the ADS1232 are closest to the capacitors (Fig. 25). Insert the four wires from the load cell into the terminal block with the black wire in the GND (upper left) hole, then the green (SIG + ), white (SIG-) and red (5 V) wires. Insert one end of each of the six jumper wires into the six pin header on the ADS1232 interface PCB. Add electrical tape to the back of the PCB to prevent connections with boards underneath.

**Table 9:** Description, board labels, color coding, type of component, and orientation for load cell PCB components.

**Part description**	**Board label**	**Type**	**Orientation**
4 position terminal block	GND, SIG+, SIG, 5 V	Through hole	Any
9-pin Female Headers	ADS1232	Through hole	Any
6-pin Female Header	+5 V, +3 V, GND, SCLK, DOUT, PDWN	Through hole	Any
Ferrite Bead	FB1, FB2	SMD	Any
47 uF 0805 Capacitor	C3, C4	SMD	Any
0.1 uF 0805 Capacitor	C2, C6	SMD	Any
CAP CER 1UF 10 V X7R 0805	C1, C5	SMD	Any
10 Ohm resistor	R2	SMD	Any
113 Ohm Resistor	R1, R3	SMD	Any

#### 5.10.5 Pressure sensor

**Fig. 26:**
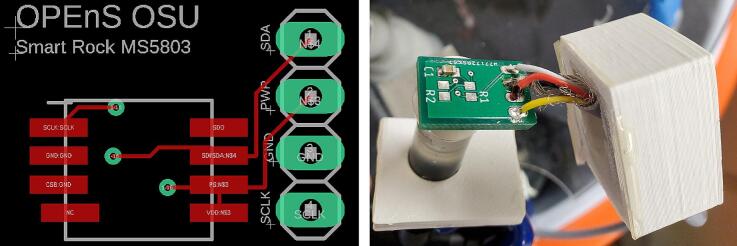
Left: Rendering of front of pressure sensor PCB showing solder pads, through holes, and silk screen. Right: Back of pressure sensor PCB with capacitor and wires soldered on, and lid and box.

Orient the pressure sensor PCB with the writing right side up for the front of the board, and place the pressure sensor so that the blue dot is at the upper left corner (Fig. 26 left). Hold the sensor in place with a binder clip or other compression device. Use small diameter solder to solder one leg onto one pad. Check the orientation of the pressure sensor, and solder the rest of the legs to the pads. Using a reflow oven or gun is not recommended for the pressure sensor. Solder the capacitor on the backside of pressure sensor PCB (Fig. 26 right). Insert one end of the 4 conductor cable through the pressure sensor box from the outside. Solder 4 wires on to the PCB (Table 10, Fig. 26 right). Conformally coat both sides of the PCB excluding the sensor itself. Feed the other end of the 4 wire cable into the electronics enclosure through the PG7 cable gland from the bottom. The wires can be directly soldered to the + 3 V, SDA and SCL through holes on the right edge and the G (for ground) in the bottom right corner of the logic module PCB, or solder seal can be used to attach jumper wires to insert into headers on the Feather M0.

**Table 10:** Wire colors by PCB label for pressure sensor.

Pressure sensor PCB	Wire color
SCLK	White
GND	Black/bare
PWR	Red
SDA	Yellow

**Fig. 27:**
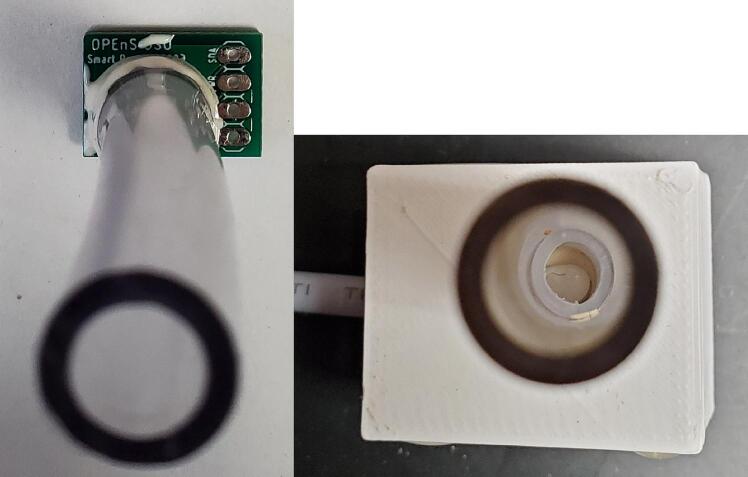
Left: Front of pressure sensor PCB with tubes epoxied on. Right: Pressure sensor inside two layers of tubes and 3D printed box.

Epoxy a 2.5 cm long piece of ¼” OD polyethylene tubing onto the outside of the sensor to create a water-tight seal, then epoxy an at least 3.5 cm long PVC tube over the top of that tube to integrate with the rest of the system (Fig. 27). Insert the tube through the 3D printed lid, coat the edge of the lid with glue or epoxy, slide the PCB into the box and clamp closed until glue or epoxy dries. Apply glue or epoxy around the tube and cable entries on the outside of the lid and box (Fig. 26).

**Fig. 28:**
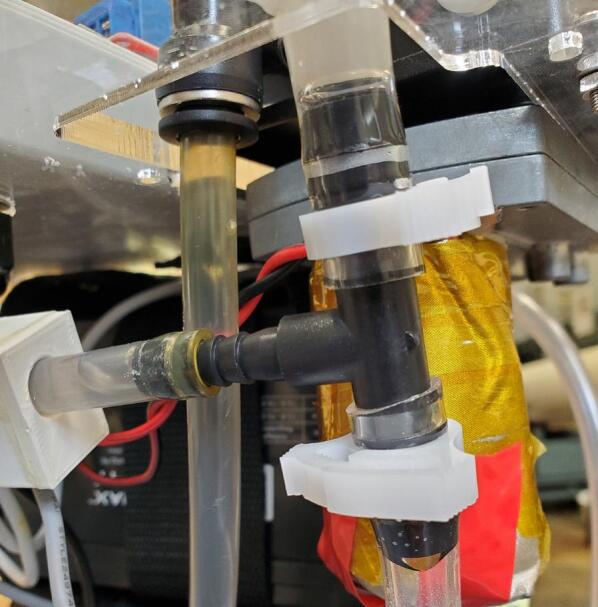
Pressure sensor assembly attached to tee on pump inlet.

Insert the other end of the tube into the perpendicular port of the barbed tee connected to the inlet side of the pump (Fig. 28). A snap grip clamp can be added to secure the tube on the barb.

#### 5.10.6 Feather M0

**Fig. 29:**
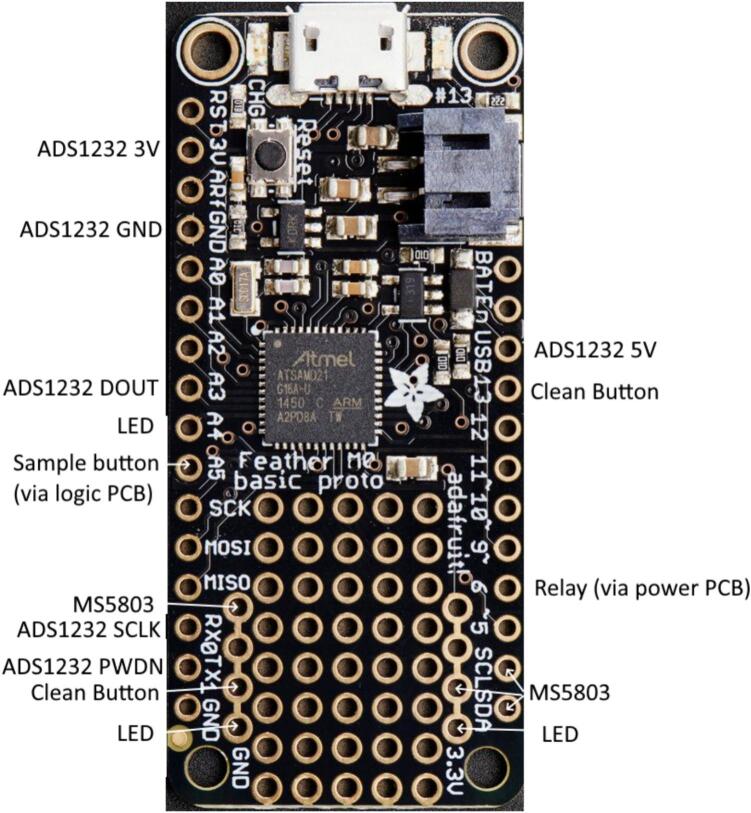
Feather M0 with labeled connections.

Solder the stacking headers onto the Feather M0 so that the long side of the pins is projecting out the bottom. Cut two sets of four headers from one of the female header sets. Solder a set of four female headers into the through holes for ground and another into the through holes for 3 V just inside the stacking headers at the end opposite the microUSB connector. Insert the stacking headers attached to the Feather M0 into the female headers attached to the logic module PCB. Following Table 11 and Fig. 29, for any connection not already soldered onto the logic PCB, connect jumper wires to the stacking header on the Feather M0. Connect any remaining 3 V (pressure sensor and LED) and ground wires (pressure sensor, clean button, LED, and sample button) to the appropriate female headers on the Feather M0. Insert the end of the micro USB cable into the port on the Feather M0.

**Table 11:** Feather M0 Connections.

Feather M0 Pin Label – 16 pin side	External Pin Label	Feather M0 Pin Label – 12 pin side	External Pin Label
3 V	ADS1232 3 V	USB	ADS1232 5 V
GND	ADS1232 GND	13	Clean button signal
A3 (also Sen1 on Logic PCB)	ADS1232 DOUT	6 (also HB1 on Power PCB)	Relay IN
A4 (also Sen2 on Logic PCB)	LED	SCL	MS5803 SCL
A5 (also switch through hole on Logic PCB)	Sample button signal	SDA	MS5803 SDA
RX_0_	ADS1232 SCLK		
TX_1_	ADS1232 PDWN		
GND	Pressure sensor GND	3.3 V	Pressure sensor PWR
GND	Clean button GND	3.3 V	LED
GND	LED GND

### 5.11 Firmware

**Fig. 30:**
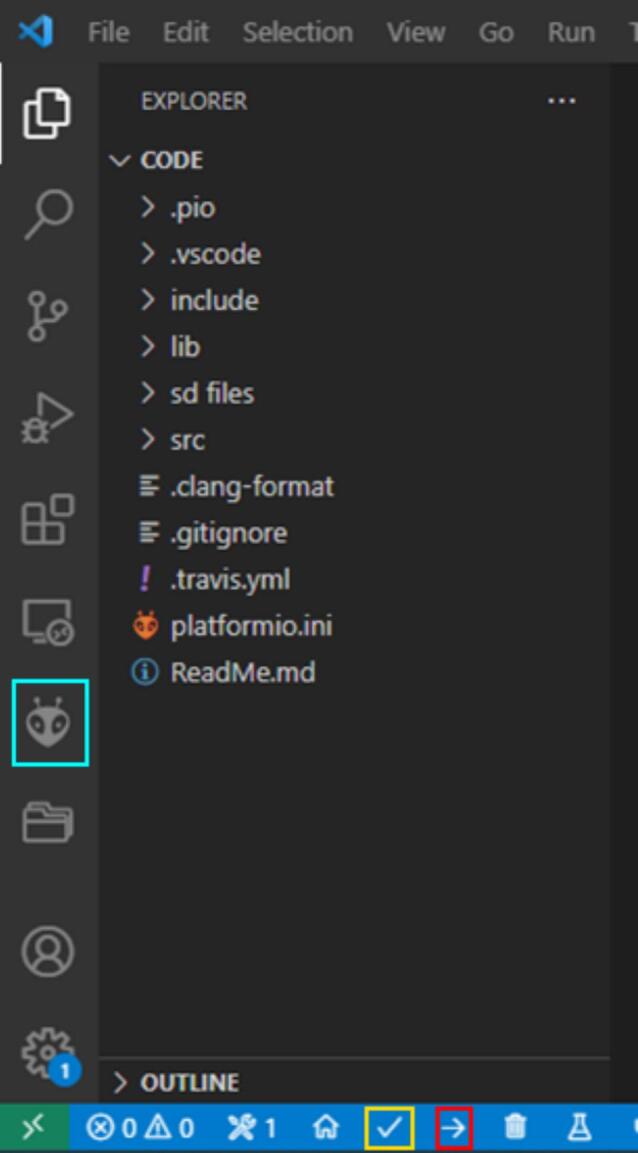
Visual Studio Code window showing code folder, sidebar, and PlatformIO toolbar. In the sidebar, a blue box has been added around the button for the PlatformIO extension. In the PlatformIO toolbar at the bottom, a yellow box has been added around the check mark for the build icon, and a red box has been added around the right arrow for the upload icon. (For interpretation of the references to color in this figure legend, the reader is referred to the web version of this article.)

Install Visual Studio Code [26]and the Platform IO extension [27]. Open the PlatformIO interface from the icon on the left sidebar to finish installation (Fig. 30), and restart Visual Studio Code as requested when finished. Click on the PlatformIO icon in the left sidebar again to load the interface. Download the code files from Zenodo. Click the “Pick a folder” button in the PlatformIO interface, and open the Code folder from the folder downloaded from Zenodo. Wait for PlatformIO to configure the project. Use the check mark in the PlatformIO toolbar to build the code (Fig. 30). PlatformIO will automatically install library dependencies based on the list in the platformio.ini file in the folder and compile the code.

Connect the Feather M0 to the computer with a microUSB cable. In quick succession, double press the Reset button on the Feather M0 to put it into bootloader mode. Click on the upload icon in the PlatformIO toolbar at the bottom of the VS Code window (Fig. 30). VS Code should automatically find the correct USB port and upload the code.

### 5.12 Calibration

The code includes coefficients, factor and offset, for converting the ADC output values to mass on lines 19 and 20 of the LoadCell.hpp in the src/Components folder. These coefficients are only used when the load cell is initially setup each time the device is turned on. For all later calculations, the coefficients on the microSD card are loaded into the microcontroller and used. The provided values are for the devices used for validation.

**Fig. 31:**
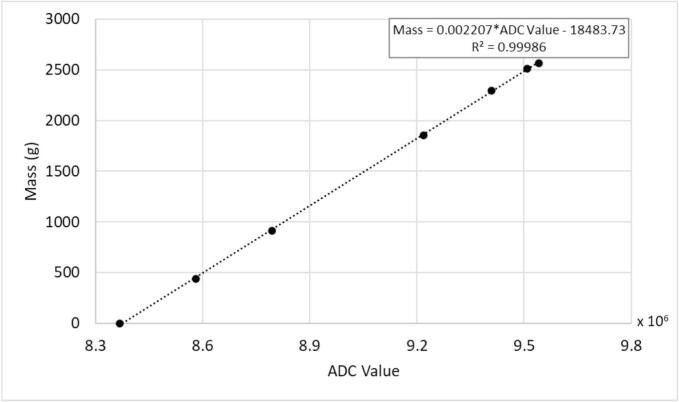
Example plot with calibration data for a sampler.

To derive the correct coefficients for each new device, start by ensuring an empty bag is attached below the load cell and use a microUSB cable to connect the microcontroller to a computer. Using a program such as the Arduino IDE or Visual Studio Code, connect to the port the microcontroller is on, and open a serial monitor. In the prompt, enter *get_load n* where n is the number of measurements to print and average across. Transfer the ADC values to a spreadsheet. Fill the bag with a known mass of liquid, attach below the load cell, and repeat measurement with the load cell. Repeat with as many masses as desired ensuring that one is at least 2000 g. Use least squares regression to derive the factor and offset such that mass = ADC value * factor + offset (Fig. 31). Update the values for the factor and offset on the microSD card. If desired, update the values in the code and re-upload to the sampler.

## 6 Operation Instructions

The auto-sampler is intended to be configured once, then deployed into the environment. Once deployed, the sampler bag should be regularly replaced with a fresh bag − normally once every 24 h. The autosampler is then reactivated and returned to the sampling site. Two interfaces, the serial monitor and the button/LED system, support this workflow after the code is uploaded to the Feather M0.

### 6.1 Fine tuning ending load criteria

If you find that the sampler is over sampling during each cycle, it may be because the first several readings of the load cell are being impacted by noise from the pump turning on, and influencing subsequent readings. In order to compensate for those initial readings, the wt_offset_load_count variable on line 64 of SampleStates.hpp can be set to calculate the expected load after the specified number of readings, and subtracted from the target weight.

### 6.2 Serial commands

The serial monitor commands change settings such as the total number of samples and the time between samples. We tested the autosampler with the Arduino IDE serial monitor application. Although all serial monitor applications will work, some may require further configuration and should be operating at 9600 baud. While the Feather M0 is connected via USB to a computer, choose the appropriate port for the Feather M0, and open the serial monitor. Commands available for modify the device behavior are listed in Table 12, Table 13 using the format command_name parameters. For example, sample_idle_time 210 s sets the idle time for the Sample state machine to 210 s.

**Table 12:** Basic commands (to be used before deployment).

**Command**	**Parameters**	**Meaning**
state_read	None	Prints all the information regarding the current parameters of the machine in JSON format.
sample_no_runs n	n: number of sample cycles	Sets the number of cycles to n before waiting for further user instruction, for example: sample_no_runs 24 Would run 24 samples of specified mass with idle time between (commands below) before waiting for the user.
sample_sample_mass g	g: sample quantity in grams	Sets the target weight to g grams.
sample_idle_time t	t: idle time in seconds	Sets the amount of time for the start of a sample cycle after the completion of the last cycle.
sample_setup_time t	t: time in seconds	Sets the amount of time between pressing the button and beginning the pressure tare routine, does not repeat. To be used to physically place the device in the environment.
sample_flush_time t	t: time in seconds	Sets the amount of time the machine is in the flush state during a sample cycle. Should be set relative to height of sampler above the water source.
clean_sample_time t	t: time in seconds	Sets the amount of time the machine is in the sample state during a clean cycle.
clean_idle_time t	t: time in seconds	Sets the amount of time the machine is in the idle state during a clean cycle.
clean_flush_time t	t: time in seconds	Sets the amount of time the machine is in the flush state during a clean cycle.

**Table 13:** Advanced commands.

**Command**	**Parameters**	**Meaning**
sample_button_press	None	Same as pressing the sample button.
clean_button_press	None	Same as pressing the clean button.
halt	None	Halts all state machines.
sample_halt	None	Halts the sample state machine.
clean_halt	None	Halts the clean state machine.
get_load n	n: number of measurements to average	Prints the current load given by the load cell.
tare_load n	n: number of measurements to average	Sets tare value to current mass.
get_tared_load n	n: number of measurements to average	Prints the current load given by the load cell, less the tare value, which is determined in the sample state.
get_pressure	None	Prints the current pressure reading of the MS5803 sensor.
get_temperature	None	Prints the current temperature reading of the MS5803 sensor.
sample_sample_time t	t: time in seconds	Sets the maximum amount of time the machine will spend sampling. In a successful sample this time would not be reached: instead, the desired load would be reached first. This prevents the machine from spending forever trying to sample when it is stuck and the pressure sensor does not detect anything.
set_time t	t: time in Unix Epoch Time	Sets the RTC to time t. Unix Epoch Time is the number of seconds since January 1, 1970.
get_time	None	Prints the RTC time in Unix Epoch Time.
shift_manip v s	v: 3 for flush, 2 for bag s: 1 for open, 0 for closed	Open and close ball valves
pump_on	None	Turn on the pump
pump_off	None	Turn off the pump
min_pressure	None	Set value for minimum allowable pressure. This is overwritten by the pressure tare at startup if enabled.
max_pressure	None	Set value for maximum allowable pressure. This is overwritten by the pressure tare at startup if enabled.
load_cell_offset n	n: value for offset	Overwrites the load cell offset on the microSD card.
load_cell_factor n	n: value for factor	Overwrites the load cell factor on the microSD card.
mem	None	Prints the remaining memory in bytes.

The required time to fully fill the system tubing with new sample water, or flush time (sample_flush_time), is a function of the tube length. For example, in testing 1.2 m required 5 s; 3 m required 35 s; and 5 m required 40 s. The default value in the code is 40 s.

### 6.3 Button use

Pressing the sample button will start the sampling protocol after a user-determined “setup” window of time (sample_setup_time command, Fig. 18). Pressing the clean button will start the cleaning protocol. Pressing the same button again will halt the protocol.

### 6.4 Deployment

Open the electronics enclosure, remove the microSD card, insert into a computer, and open the state.js file to verify that all sampling and calibration parameters are appropriate. Reinstall the microSD card, and insert the barrel jack into the socket on the logic board. Close and secure the electronics enclosure with screws. Attach appropriate length inlet and outlet tubes to the sampler using the quick disconnect and push to connect, respectively. A weighted intake on the inlet tube can be helpful for keeping it in place. Ensure that a bag is connected to the quick disconnect below the load cell rotated such that the sides of the bag are not touching anything. Set the acrylic circle to its lowest position and ensure the inlet and outlet tubing will not touch the bag as it fills. Press the sampling button, verify the LED turns green, and screw on the cooler lid. Put the free ends of the inlet and outlet tubes in the liquid source, and setup the sampler in a stable position above. Return after the expected time period for all samples to remove the sampler. Raise the acrylic circle to its full height and carefully disconnect the bag by pressing on the grey tab on the quick disconnect. Process the sample per the appropriate protocol. Open the electronics enclosure, remove the microSD card, insert into a computer, and copy over the Data.csv file for a log of all sampling events.

## 7 Validation and characterization

### 7.1 Power budget

The sampler exists in two general power conditions during typical operations: sampling (i.e. all electronics and pump on) or idle (i.e. pump off and electronics on). The total available energy for each deployment is 7.2 Ah at 12 V or 86.4 Wh. Current draw during sampling is up to 1.3 A which corresponds to 15.6 W at the 12 V supplied by the battery, and current draw when the sampler is idle is up to 0.033 A which corresponds to 0.396 W at the 12 V supplied by the battery. The amount of time associated with each power condition is a function of hard coded transition times, pumping speed, and several user definable parameters: sample flush time, sample volume, sample idle time (Fig. 4). The total power usage for a single deployment is also a function of the user definable number of sample runs. Three example calculations are provided in Table 14 with a total volume of 2 L (to account for sample sizes larger than targeted value) for multiple samples, and 2.9 L for a single sample. For the calculation, the flush time is set to 40 s, and that pumping speed is assumed to be 1 g/s for 10 g and 1.6 g/s for larger volumes.

Sampleruntime=prep+sample

10 g samples

$$=\left(5+40+5+5\right)+10=65seconds$$

$$=65s*\frac{1min}{60s}*\frac{1hr}{60min}=0.018hour$$

100 g samples $=\left(5+40+5+5\right)+60=115seconds$

$$=115s*\frac{1min}{60s}*\frac{1hr}{60min}=0.032hour$$

2900 g sample

**Table 14:** Values for example power budget calculations.

Sample size (g)	Number of samples	Sampling run time period (h)	Total sampling time period (h)^a^	Total sampling energy usage (Wh)^b^	Available idle energy (Wh)^c^	Maximum idle time (h)^d^	Maximum deployment time (days)
10	200	0.018	3.62	56.5	29.9	75.5	6.6
100	20	0.032	0.98	15.3	71.1	179.5	15.0
2900	1	0.52	0.52	8.1	78.3	197.7	16.5

a Includes 0.02 h one time pressure tare step.

b Total Sampling Energy Usage = Total sampling time * Power usage per sample run (15.6 W).

c Available idle energy = Total Energy (86.4 WH) – Total Sampling Energy Usage.

d Maximum idle time = Available idle power / Power usage while idle (0.396 W).

= (5 + 40 + 5 + 5) + 1812.5 = 1867.5 s

$$=1867.5s*\frac{1min}{60s}*\frac{1hr}{60min}=0.52hour$$

### 7.2 Sample size

Two prototypes of the sampler were tested at heights above water sources ranging from 0.65 to 4.75 m (Table 15). There was little difference in performance between heights for 10 g and 100 g targets, and for 30 s to 1-hour intervals between samples, so all results are lumped. In all cases, the mean difference in sample weight from target weight was −5.7 gor less, and the standard deviation in sample weight from target weight was 6.5 g or less (Table 15).

**Table 15:** Mean and standard deviation of samples with a 10 or 100 g target for two sampler prototypes.

	Sampler 1			Sampler 2	
Height above water source (m)	0.65––0.95	2.75––3.1	4.4–––4.75	0.65	4.75
Count	30	115	124	35	2
Difference from target weight − mean (g)	−4.4	−1.9	−5.7	2.8	−0.1
Difference from target weight − standard deviation (g)	6.5	3.9	4.4	2.6	5.8

### 7.3 Pumping speed

Pumping speed was tested at 0.65 to 4.75 m heights above water supplies with the designed nominal input voltage of 12 V, and the maximum pump allowed voltage of 24 V. At 0.65 to 0.95 m height and 12 V power supply, the mean pumping rate during sampling was 23.2 g/s or 0.32 m/s in the 3/8″ inside diameter tubing of the inlet. At 0.95 m height and 24 V power supply, the mean pumping rate during sampling was 39.2 g/s or 0.55 m/s in the 3/8″ inside diameter tubing of the inlet. At 2.75 to 4.75 m height and 12 V power supply, pumping rate during sampling was 2 g/s or 0.03 m/s in the 3/8″ inside diameter tubing of the inlet.

### 7.4 Pressure sensor performance

**Fig. 32:**
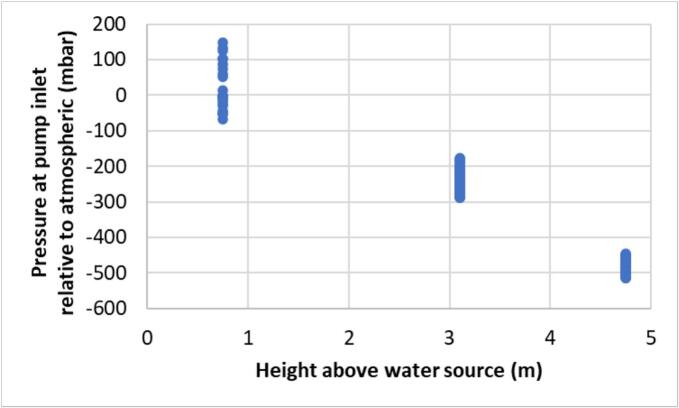
Pressure at the pump inlet at the end of each sampler cycle relative to the height of the sampler above the water source.

The pressure sensor was evaluated at the three heights (0.75 m, 3.1 m, and 4.75 m) with measurements taken at the end of each sampling cycle (Fig. 32). Atmospheric pressure ranged from 999 to 1016 mbar during the measurements. As expected, there is an inverse linear relationship between pressure and height above the water source.

**Fig. 33:**
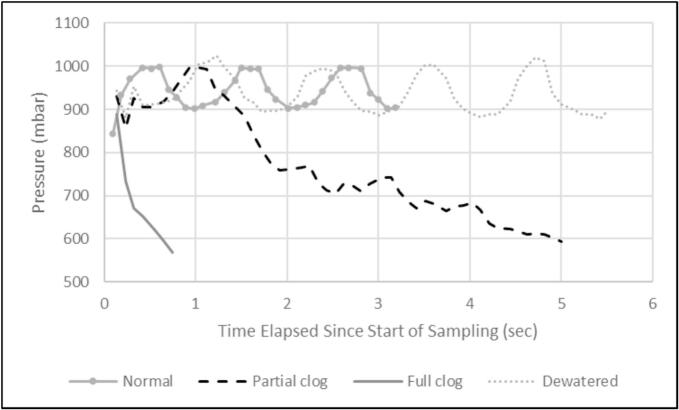
Pressure during sampling with the inlet tubing dewatered, fully submerged and unclogged (normal), partially and fully clogged.

The pressure and time stopping criteria were tested in circumstances simulating clogging and dewatering at 0.75 m height. The sampler was allowed to run through a normal setup with an average pressure of 945 mbar, and an acceptable range of 595 to 1295 mbar, and a 100 g sampling cycle with the intake fully submerged and unblocked (Fig. 33). A partial clog was then simulated during the next cycle by completely bending over the inlet tubing at the start of the sample which triggered the stop of sampling after 5 s due to low pressure (Fig. 33). A full clog was simulated in the next cycle by completely blocking the inlet tube entrance at the start of the sample which triggered the stop of sampling after 0.8 s due to low pressure (Fig. 33). During the final cycle, the end of the inlet tubing was removed from the water source at the start of the sample and sampled was stopped due to time after 5.5 s.

### 7.5 Temperature

**Fig. 34:**
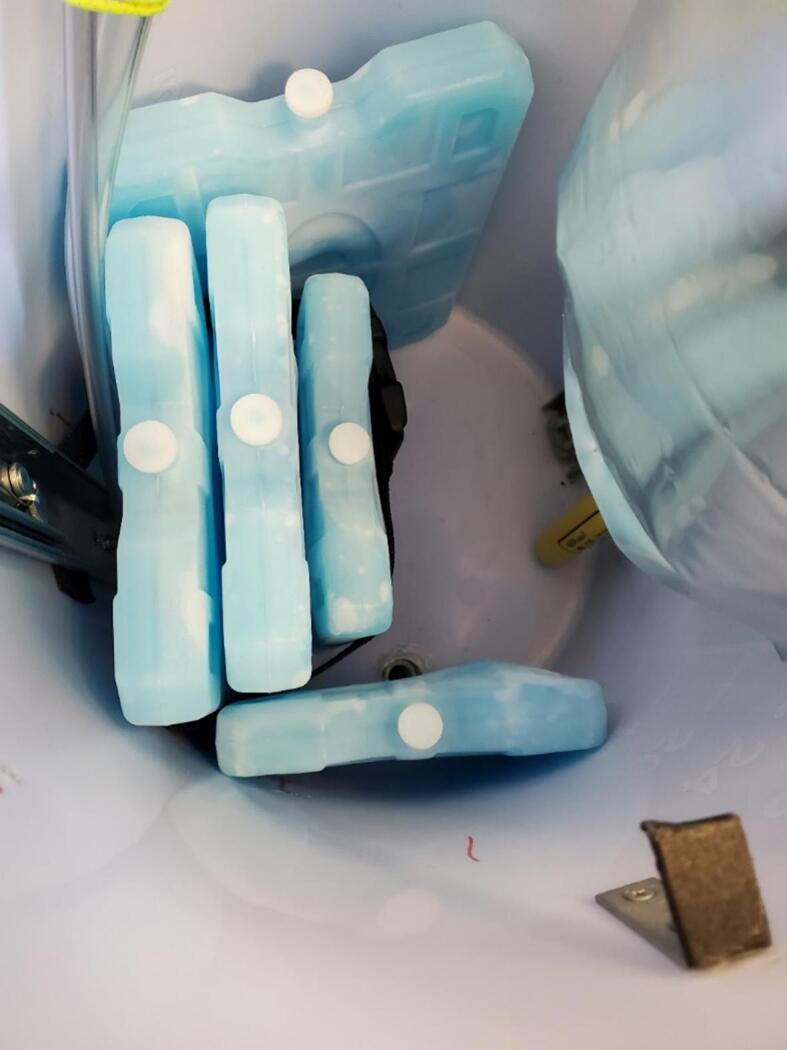
Ice packs and RBR-Solo T placed in the cooler. Note bag was lowered into place prior to start of testing.

**Fig. 35:**
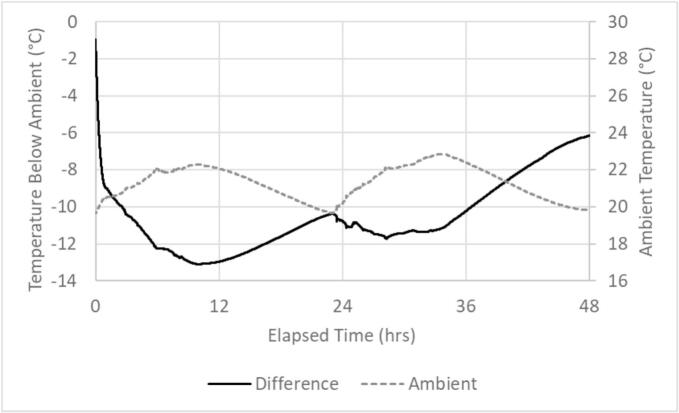
Temperature inside the sampler relative to outside the cooler with 3 L of water in the cooler at an initial temperature of 18 °C.

The sample bag was filled with 18 °C water and attached below the load cell, and three large and two medium frozen ice packs were placed sitting upright around the bag (Fig. 34). The bag was lowered into place, and an RBR-Solo T logger was placed next to the bag. The lid was screwed onto the cooler and another RBR-Solo-T logger was placed on the lid for capturing ambient temperature. The minimum temperature reached in the cooler was 13 °C below ambient after 11 h and increased only approximately 2 °C over the next 13 h (Fig. 35). The inside temperature was 6 °C below ambient after 48 h. Ambient temperature ranged from 19.7 °C to 22.8 °C.

## 8 Conclusion

Development of low-cost sampling devices leads to improved monitoring, and ability to test for presence of viruses and other pathogens in wastewater. This Autosampler design is low-cost (approximately $1000) compared to other samplers on the market. As an added benefit, this design’s serial monitor permits easy change of the sampler’s operation status using the Arduino program.

## CRediT authorship contribution statement

**Hadi A. Al-agele:** Writing – review & editing, Writing – original draft, Visualization, Validation, Software, Project administration, Methodology, Investigation, Formal analysis, Data curation, Conceptualization. **Bao D. Nguyen:** Software, Methodology, Formal analysis. **Liam P. Zimmermann:** Writing – original draft, Software, Methodology. **Gurpreet Singh:** Writing – original draft, Validation, Methodology, Investigation, Formal analysis, Data curation. **Cara Walter:** Writing – review & editing, Writing – original draft, Visualization, Validation, Project administration, Methodology, Investigation, Formal analysis, Data curation, Conceptualization. **Chet Udell:** Visualization, Validation, Supervision, Project administration, Methodology, Investigation, Conceptualization. **John S. Selker:** Writing – review & editing, Visualization, Validation, Supervision, Software, Resources, Project administration, Methodology, Investigation, Funding acquisition, Formal analysis, Data curation, Conceptualization.

## Declaration of competing interest

The authors declare that they have no known competing financial interests or personal relationships that could have appeared to influence the work reported in this paper.
