# Supplementary material for: Low-cost composite autosampler for wastewater sampling
Source: HardwareX. 2025 Mar 1;21:e00631. doi: 10.1016/j.ohx.2025.e00631 (PMC11925591; doi:10.1016/j.ohx.2025.e00631)
Supplement: Supplementary Data 1 [file mmc1.pdf]

Designator	Component	Number	Cost per unit $	Minimum purchase quantity	Cost $ per sampler	Source of materials	Material type
**Enclosures - Device**							
Cooler	10 gal watercooler	1	$49.89	1	$49.89	Homedepot	Other
Tube holder	3D printed tube holder	1	$0.19	1	$0.19	Zenodo	Polymer
Epoxy	Loctite 1919324 Marine Epoxy	1	$7.74	1	$7.74	Amazon	Polymer
Corner Brace	2 in galvanized corner brace with screws	4	$1.62	4	$6.46	Home Depot	Metal
Star drive screw	3/4" 18-8 stainless steel screw	8	$0.42	25	$3.36	Amazon	Metal
1/2" PVC coupler	1/2" schedule 40 PVC coupler	1	$0.67	1	$0.67	Home Depot	Polymer
Buckles	Adjustable Quick Side Release Buckles	2	$1.05	4	$2.1	Amazon	Polymer
Webbing	Heavyweight Polypropylene Webbing 2 inch	4 ft	$1.22	30 ft	$4.88	Amazon	Polymer
Drawer slides	Heavy Duty Drawer Slides 14 inch	2	$28.40	2	$56.80	Amazon	Composite
L bracket	Aluminum 90° Angle - 3.5" height, 1/4" thick	1	$21.27	1	$21.27	McMaster Carr	Metal
5 mm washers	5 mm Stainless Steel Washers	8	$0.31	4	$2.50	Home Depot	Metal
Flathead bolt	M5, 16 mm long bolt	6	$0.24	100	$1.44	McMaster Carr	Metal
Lock nut	M5 lock nut	6	$0.63	2	$3.75	Home Depot	Metal
Acrylic Sheet	Acrylic - clear 24" x 24", 1/8"	1	$26.08	1	$26.08	McMaster Carr	Polymer
Large ice pack	Reusable Ice Pack, 1 Large	3	$7.38	4	$22.14	Amazon	Composite
Medium ice pack	Reusable Ice Pack, 1 Medium	2	$3.79	4	$7.58	Amazon	Composite
Cable with carabiner	Stainless Steel Safety Cable Lock	3	$2.40	4	$7.2	Amazon	Metal
1/8" wire rope	Stainless steel wire rope, 1/8"	10 ft	$0.54	33 ft	$5.4	Amazon	Metal
Battery	12 Volt 7.2 Ah SLA battery	1	$17.50	1	$17.50	Amazon	Other
Quick-Disconnect Terminal	Quick-Disconnect Terminal	2	$1.08	1	$2.16	McMaster Carr	Composite
Paracord	1/8" x 50 ft paracord	5 ft	$0.11	50 ft	$0.55	Home Depot	Polymer

**Hydraulics - Pump**							
Peristaltic Pump	Peristaltic Pump DC 12Vto 24V	1	$49.99	1	$49.99	Ebay	Other
Pump tubing	Hi-Temp Silicone Rubber Tubing	1 ft	$1.39	10 ft	$1.39	McMaster Carr	Polymer
Tube to hose stem	3/8" to 3/8" Tube To Hose Stem	1	$2.39	10	$2.39	Home Depot	Polymer
Cable tie	Cable tie, 0.1" width, 4" length	2	$0.04	100	$0.08	McMaster Carr	Polymer
Elbow push-to-connect	3/8 OD x 3/8 OD elbow push-to-connect	1	$1.30	10	$1.30	Amazon	Composite

Designator	Component	Number	Cost per unit $	Minimum purchase quantity	Cost $ per sampler	Source of materials	Material type
Elbow barbed	Barbed elbow, 3/8" OD	1	$1.02	1	$1.02	McMaster Carr	Polymer
3/8" tubing	Soft ND-100-65 Tygon PVC Tubing	1	$93.00	25 ft	$93.00	McMaster Carr	Polymer
Small snap-grip clamps	Snap-Grip Clamp 29/64" to 9/16" Clamp ID	2	$0.46	25	$0.92	McMaster-Carr	Polymer
Faraday Fabric	EMF shielding Film	1	$23.00	44"W x 36"L	$23.00	Amazon	Other
Electrical tape	Electrical tape	1 ft	$5.98	66 ft	$5.98	Home Depot	Polymer
M3 bolt - 16 mm	16mm M3 screw low profile	9	$0.13	100	$1.17	McMaster Carr	Metal
M3 washer - 9 mm	18-8 Stainless Steel Oversized Washer M3	13	$0.05	100	$0.65	McMaster Carr	Metal
M3 nuts	M3 nuts stainless steel	9	$0.06	50	$0.54	McMaster Carr	Metal
Barbed tee fitting	Barbed tee fitting, 3/8" OD	1	$1.40	10	$1.40	McMaster Carr	Polymer
Quick-Disconnect Plug to 3/8" barbed	Plug, 1/4 Size, for 3/8" Barbed Tube ID	1	$4.11	1	$4.11	McMaster Carr	Polymer
Quick-Disconnect Socket to 3/8" barbed	Socket, 1/4 Size, for 3/8" Barbed Tube ID	1	$5.58	1	$5.58	McMaster Carr	Polymer
Polyethylene tubing	Polyethylene Tube, Shore D: 45, 1/4 in ID	1	$4.10	10 ft	$4.10	Grainger	Polymer
Tee push-to-connect	3/8" OD Tee push-to-connect	1	$1.10	10	$1.10	Amazon	Composite
**Hydraulics - Valves**							
Motorized Ball valve - normally closed	Motorized Ball valve 1/4" NPT - auto return	2	$38.29	1	$76.58	Amazon	Other
PTFE tape	PTFE tape	1	$0.98	260 in.	$0.98	Home Depot	Polymer

Straight 3/8” push to connect to ¼ NPT	3/8"OD push to connect x 1/4 Inch NPT	2	$1.80	10	$3.6	Amazon	Composite
Barbed 3/8" elbow to 1/4" NPT Male	Barbed elbow 3/8" ID x 1/4 NPT Male	1	$2.73	1	$2.73	McMaster Carr	Polymer
Elbow 3/8" push-to-connect to 1/4" NPT	3/8 OD elbow push-to-connect to 1/4 NPT	1	$1.88	5	$1.88	Amazon	Composite
Valve bracket	3D printed valve bracket	2	$0.41	1	$0.81	Zenodo	Polymer
Large snap-grip clamps	Snap-Grip Clamp 37/64" to 41/64" Clamp ID	1	$0.49	25	$12.17	McMaster Carr	Polymer
PVC tubing	PVC Firm tubing 1/4 ID 3/8 OD	1 ft	$16.80	25 ft	$16.80	McMaster Carr	Polymer
Straight 3/8" push to connect fitting	Straight 3/8" push to connect fitting	1	$3.81	1	$3.81	McMaster Carr	Composite
**Hydraulics - Sample bag**							
Mylar Bag	3L Mylar Bag	1	$0.89	250	$221.80	IMPAK	Other
Threaded valved coupler	Coupler, Push In, Acetal, White	1	$6.08	1	$6.08	CPC	Polymer
Eye bolt and nut	Eye bolt and nuts	1	$0.69	2	$1.38	Home Depot	Metal

**Designator**	**Component**	**Number**	**Cost per unit $**	**Minimum purchase quantity**	**Cost $ per sampler**	**Source of materials**	**Material type**
Barbed elbow coupling insert	Coupler, Push In, Acetal, White	1	$1.40	1	$1.40	CPC	Polymer
1/16" wire rope	1/16" wire rope	1	$0.98	1 ft	$0.98	Home Depot	Metal
Compression sleeve	1/16" Wire Rope Compression Sleeve	1	$0.97	10	$9.69	McMaster-Carr	Metal
**Load Cell Mechanical**							
M5 bolt	M5 bolt, 40 mm	1	$1.38	2	$1.38	Home Depot	Metal
Large Washer	18 mm OD M5 washer	2	$0.14	100	$0.24	McMaster Carr	Metal
Neoprene washer	1/4" thick neoprene washer	2	$0.69	4	$1.38	Home Depot	Polymer
Spacer	Spacer - 7 mm	1	$1.76	1	$1.76	McMaster Carr	Metal
M5 nut	M5 stainless steel nut	1	$0.63	2	$0.63	Home Depot	Metal
**Enclosures - Electronics**							
Electronic Box	Polycarbonate waterproof box	1	$5.88	1	$5.88	Arrow	Polymer

RGB LED	WS2812B LED	1	$0.16	100	$0.16	Amazon	Composite
LED box	LED 3D printed box	1	$0.04	1	$0.04	OPEnS	Polymer
LED lid	LED 3D printed lid	1	$0.05	1	$0.05	OPEnS	Polymer
3 wire cable	3 wire cable	5 ft	$0.31	100 ft	$1.55	Amazon	Composite
Super glue	Gorilla Super Glue Gel	1	$6.84	1	$6.84	Amazon	Polymer
Push button	Momentary Push button waterproof	2	$2.20	5	$4.40	Amazon	Composite
2 pin JST wire pairs	2 pin JST wire pairs	4	$1.60	5	$6.4	Amazon	Composite
Small heat shrink	Small heat shrink	4	$0.01	580	$0.04	Amazon	Polymer
Solder seal connectors	Solder Seal Wire Connectors 22-18 AWG	4	$0.20	50	$0.80	Amazon	Composite
PG7 cable gland	PG7 cable gland	1	$1.52	1	$1.52	Mouser	Polymer
PG13.5 cable gland	PG13.5 cable gland	1	$1.89	1	$1.89	Mouser	Polymer
Power plug	Right Angle Power Plug Jack to Bare Wire	1	$1.60	10	$15.99	Amazon	Composite
microUSB cable	Right angle microUSB cable	1	$5.58	1	$5.58	~~DigiKey~~	Other
Stranded wire: black, red, white, green	22 AWG Gauge Stranded Wire, 6 colors	1	$1.25	6 rolls at 5 ft	$7.50	Amazon	Composite
**Power Module PCB Components**							
Power module PCB	Power module mini PCB	1	$2.30	5	$2.30	Zenodo	Composite
Logic level converter	Sparkfun logic level shifter	1	$2.95	1	$2.95	Sparkfun	Composite

**Designator**	**Component**	**Number**	**Cost per unit $**	**Minimum purchase quantity**	**Cost $ per sampler**	**Source of materials**	**Material type**
12 pin angle female header	12 pins angle female header	1	$0.80	1	$0.80	DigiKey	Composite
32K resistor	Resistor 32K Ohm 3.3mm	1	$0.10	1	$0.20	DigiKey	Other
470uF capacitor	Capacitor 470uF SMD 16V	1	$0.51	1	$0.51	DigiKey	Other
100 nF capacitor	Capacitor 100nF SMD	4	$0.12	1	$0.48	DigiKey	Other
Flyback Diode	Diode Standard 100V 150 mA	2	$0.15	1	$0.30	DigiKey	Other

Shift register	IC Power 8-bit shift Register 20-SOIC	1	$1.42	1	$5.68	DigiKey	Other
Relay	1 Channel DC 5V Relay Module	1	$1.75	1	$6.99	Amazon	Other
Standoff	M3 standoff	1	$2.17	1	$2.17	McMaster Carr	Metal
M3 6 mm bolt	M3 6 mm bolt	1	$0.05	50	$2.51	Grainger	Metal
Rectifier Diode	Rectifiers Diode, DO-41, 50V, 1A	1	$0.40	1	$0.40	Mouser	Other
**Logic Module Components**							
Logic pcb	Logic Module PCB	1	$2.30	5	$11.50	Zenodo	Composite
M0 female header	Feather M0 header female	1	$0.95	1	$0.95	Adafruit	Composite
Angle male header	Angle male header 2.54 mm	12	$0.02	1	$0.24	DigiKey	Composite
MicroSD mount	MicroSD card mount SMD - Hinged type	1	$1.16	1	$1.16	DigiKey	Metal
MicroSD card	16 GB microSD card with adapter	1	$7.99	1	$7.99	Amazon	Other
Coin cell battery holder	12mm Battery holder	1	$1.30	1	$1.30	DigiKey	Metal
Coin cell battery	CR1220	1	$0.52	1	$0.52	~~Grainger~~	Other
Barrel jack	Power Barrel Connector Jack Surface Mount	1	$1.32	1	$1.32	DigiKey	Composite
Switch	Slide switch DPDT 300MA 6V	1	$0.55	1	$0.55	DigiKey	Composite
RTC	RTC I2C 16-SOIC DS3231	1	$9.78	1	$9.78	DigiKey	Other
P-channel MOSFET	P-channel MOSFET 130mA	1	$0.27	1	$0.27	DigiKey	Other
Not-Gate	Inverter IC 1 Channel - SOT-23-5	1	$0.34	1	$0.34	DigiKey	Other
D Flipflop	D Flip Flop SOT23-6	1	$0.37	1	$0.37	DigiKey	Other
Zener Diode	Zener Diode 4.3 V 800 mW - Surface Mount	1	$0.44	1	$0.44	DigiKey	Other
Diode Schottky	Diode Schottky 1A (DC) Surface Mount	1	$0.39	1	$0.39	DigiKey	Other
Inductor	Inductor 10uH 2,7A	1	$1.69	1	$1.69	DigiKey	Other
Linear regulator	Buck regulator	1	$2.77	1	$2.77	TI	Other

Designator	Component	Number	Cost per unit $	Minimum purchase quantity	Cost $ per sampler	Source of materials	Material type
Red LED	KingBright Red LED low current 0805	1	$0.51	1	$0.51	DigiKey	Other
Yellow LED	KingBright Yellow LED low current 0805	1	$0.51	1	$0.51	DigiKey	Other
3K resistor	Resistor 3K Ohm resistor 1%	1	$0.10	1	$0.10	DigiKey	Other
6.2K resistor	Resistor 6.2K Ohm 1% 1206	1	$0.10	1	$0.10	DigiKey	Other
10K resistor	Resistor 10k Ohm 1% 1206	3	$0.10	1	$0.30	DigiKey	Other
10K through hole resistor	10k Ohm through hole resistor 1%	2	$0.10	1	$0.20	DigiKey	Other
39K resistor	Resistor 39K Ohm 5% 1206	1	$0.10	1	$0.10	DigiKey	Other
100K resistor	Resistor 100K Ohm 1% 1206	1	$0.10	1	$0.10	DigiKey	Other
300K resistor	Resistor 300K Ohm 0.1% 1206	1	$0.66	1	$0.66	DigiKey	Other
1M resistor	Resistor 1M Ohm 0.1% 1206	1	$0.62	1	$0.62	DigiKey	Other
0.1uF 1206 capacitor	Capacitor 0.1uF 100V 1206	5	$0.19	1	$0.95	DigiKey	Other
10uF 1206 capacitor	Capacitor 10uF 25V 1206	1	$0.22	1	$0.22	DigiKey	Other
47uF 1206 capacitor	Capacitor 47uF 10V 1206	1	$0.69	1	$0.69	DigiKey	Other
30k ohm resistor	30k ohm resistor 5%	1	$0.10	1	$0.10	DigiKey	Other
**Load Cell Circuit**							
ADS1232	24-bit ADC	1	$16.99	1	$16.99	Amazon	Other
ADS1232 interface PCB	ADS1232 interface PCB	1	$3.83	3	$11.49	Zenodo	Other
4 position terminal block	Terminal Block 4 Position Top Entry 2.54mm	1	$1.52	1	$1.52	DigiKey	Composite
Female Header Set	12-pin and 16-pin Female Header Set	1	$0.95	1	$0.95	Adafruit	Composite
Ferrite Bead	Ferrite Bead 300 Ohm 0805 1LN	2	$0.19	1	$0.38	DigiKey	Other
47 uF 0805 Capacitor	Capacitor Ceramic 47uF 10V X5R 0805	2	$1.25	1	$2.50	DigiKey	Other
0.1 uF 0805 Capacitor	Capacitor Ceramic 0.1uF 10V X7R 0805	2	$0.18	1	$0.36	DigiKey	Other
1uF X7R 0805 Capacitor	Capacitor ceramic 1uF 10V X7R 0805	2	$0.29	1	$0.58	DigiKey	Other

10 Ohm resistor	Resistor SMD 10 Ohm 0.1% 1/4W 0805	1	$0.96	1	$0.96	DigiKey	Other
113 Ohm Resistor	Resistor SMD 113 Ohm 0.1% 1/4W 0805	2	$0.69	1	$1.38	DigiKey	Other
Male Jumper Wires 150 mm	Male Jumper Wires 150 mm	1	$0.10	20	$1.95	Adafruit	Composite
Load Cell	Micro Load Cell (0-5Kg)	1	$7.00	1	$7.00	Phidgets	Other
**Pressure Sensor**							

**Designator**	**Component**	**Number**	**Cost per unit $**	**Minimum purchase quantity**	**Cost $ per sampler**	**Source of materials**	**Material type**
Pressure sensor PCB	Pressure sensor PCB	1	$0.50	3	$1.50	Zenodo	Composite
Pressure Sensor	Pressure Sensor MS5803-02BA	1	$10.72	1	$10.72	DigiKey	Other
0.1 uF Capacitor	0.1 uF Capacitor 1206	1	$0.20	1	$0.20	DigiKey	Other
Pressure sensor box lid	Pressure sensor box lid	1	$0.07	1	$0.07	Zenodo	Polymer
Pressure sensor box	Pressure sensor box	1	$0.18	1	$0.18	Zenodo	Polymer
4 conductor shielded wire	4 conductor shielded wire	1	$5.98	1 m	$5.98	DigiKey	Composite
1/4" OD polyethylene tube	Crack-Resistant Polyethylene Firm Tubing	1 ft	$3.25	25 ft	$3.25	McMaster Carr	Polymer
Conformal coating	Conformal coating	1	$17.00	1	$17.00	Amazon	Other
**Feather M0**							
Feather M0	Feather M0 Basic Proto	1	$19.95	1	$19.95	Adafruit	Other
M0 stacking header	Feather M0 header stacking	1	$1.25	1	$1.25	Adafruit	Composite
M0 female header	Feather M0 header female	1	$0.95	1	$0.95	Adafruit	Composite
Total cost per sampler					~$1000		
