# Supplementary material for: Low-cost composite autosampler for wastewater sampling
Source: HardwareX. 2025 Mar 1;21:e00631. doi: 10.1016/j.ohx.2025.e00631 (PMC11925591; doi:10.1016/j.ohx.2025.e00631)
Supplement: Supplementary Data 2 [file mmc2.pdf]

Design files

Design file name	File type	Open-source license	Location of the file
Acrylic circle	Dxf	CERN-OHL-S	https://doi.org/10.5281/zenodo.8333206
ADS1232 interface	Brd	CERN-OHL-S	https://doi.org/10.5281/zenodo.8333206
ADS1232 interface	Sch	CERN-OHL-S	https://doi.org/10.5281/zenodo.8333206
Enclosure bottom	Dxf	CERN-OHL-S	https://doi.org/10.5281/zenodo.8333206
Enclosure insert	Dxf	CERN-OHL-S	https://doi.org/10.5281/zenodo.8333206
LED Box	Stl	CERN-OHL-S	https://doi.org/10.5281/zenodo.8333206
LED Lid	Stl	CERN-OHL-S	https://doi.org/10.5281/zenodo.8333206
Logic Module V3	Brd	CERN-OHL-S	https://doi.org/10.5281/zenodo.8333206
Logic Module V3	Sch	CERN-OHL-S	https://doi.org/10.5281/zenodo.8333206
MS5803	Brd	CERN-OHL-S	https://doi.org/10.5281/zenodo.8333206
MS5803	Sch	CERN-OHL-S	https://doi.org/10.5281/zenodo.8333206
Power module mini	Brd	CERN-OHL-S	https://doi.org/10.5281/zenodo.8333206
Power module mini	Sch	CERN-OHL-S	https://doi.org/10.5281/zenodo.8333206
Pressure sensor box	Stl	CERN-OHL-S	https://doi.org/10.5281/zenodo.8333206
Pressure sensor box lid	Stl	CERN-OHL-S	https://doi.org/10.5281/zenodo.8333206
Tube holder	Stl	CERN-OHL-S	https://doi.org/10.5281/zenodo.8333206
Valve bracket	Stl	CERN-OHL-S	https://doi.org/10.5281/zenodo.8333206
Application	Hpp	GNU General Public License v3.0	https://doi.org/10.5281/zenodo.8333206
Clock	Hpp	GNU General Public License v3.0	https://doi.org/10.5281/zenodo.8333206
Constants	Hpp	GNU General Public License v3.0	https://doi.org/10.5281/zenodo.8333206
Button	Cpp	GNU General Public License v3.0	https://doi.org/10.5281/zenodo.8333206
Button	Hpp	GNU General Public License v3.0	https://doi.org/10.5281/zenodo.8333206
LED	Hpp	GNU General Public License v3.0	https://doi.org/10.5281/zenodo.8333206
LoadCell	Hpp	GNU General Public License v3.0	https://doi.org/10.5281/zenodo.8333206
PressureSensor	Hpp	GNU General Public License v3.0	https://doi.org/10.5281/zenodo.8333206
Pump	Hpp	GNU General Public License v3.0	https://doi.org/10.5281/zenodo.8333206

Shell	Cpp	GNU General Public License v3.0	https://doi.org/10.5281/zenodo.8333206
Shell	Hpp	GNU General Public License v3.0	https://doi.org/10.5281/zenodo.8333206
ShiftRegister	Hpp	GNU General Public License v3.0	https://doi.org/10.5281/zenodo.8333206
StateMachine	Hpp	GNU General Public License v3.0	https://doi.org/10.5281/zenodo.8333206
CSVWriter	Hpp	GNU General Public License v3.0	https://doi.org/10.5281/zenodo.8333206
JsonEncodable Decodable	Hpp	GNU General Public License v3.0	https://doi.org/10.5281/zenodo.8333206
CleanStateMac hine	Hpp	GNU General Public License v3.0	https://doi.org/10.5281/zenodo.8333206
CleanStates	Cpp	GNU General Public License v3.0	https://doi.org/10.5281/zenodo.8333206
CleanStates	Hpp	GNU General Public License v3.0	https://doi.org/10.5281/zenodo.8333206
SampleStateMa chine	Hpp	GNU General Public License v3.0	https://doi.org/10.5281/zenodo.8333206
SampleStates	Cpp	GNU General Public License v3.0	https://doi.org/10.5281/zenodo.8333206
SampleStates	Hpp	GNU General Public License v3.0	https://doi.org/10.5281/zenodo.8333206
main	Cpp	GNU General Public License v3.0	https://doi.org/10.5281/zenodo.8333206
platformio	Ini	GNU General Public License v3.0	https://doi.org/10.5281/zenodo.8333206
state	Js	GNU General Public License v3.0	https://doi.org/10.5281/zenodo.8333206
